# Dephosphorylation of HDAC4 by PP2A-Bδ unravels a new role for the HDAC4/MEF2 axis in myoblast fusion

**DOI:** 10.1038/s41419-019-1743-6

**Published:** 2019-07-04

**Authors:** Alexandra Veloso, Maud Martin, Jonathan Bruyr, Tina O’Grady, Christophe Deroanne, Denis Mottet, Jean-Claude Twizere, Thomas Cherrier, Franck Dequiedt

**Affiliations:** 1 Interdisciplinary Cluster for Applied Genoproteomics (GIGA-R), University of Liège (ULg), Liège, Belgium; 2 GIGA-Molecular Biology of Diseases, University of Liège, Liège, Belgium; 3 Laboratory of Neurovascular Signaling, Department of Molecular Biology, ULB Neuroscience Institute, Université libre de Bruxelles (ULB), B-6041 Gosselies, Belgium; 4 Laboratory of Connective Tissues Biology, GIGA-Cancer, University of Liège, Sart-Tilman, Belgium; 5 Univ. Bourgogne Franche-Comté, INSERM, EFS BFC, UMR1098, RIGHT Interactions Greffon-Hôte-Tumeur/Ingénierie Cellulaire et Génique, F-25000 Besançon, France

**Keywords:** Cytoskeleton, Differentiation, Phosphorylation

## Abstract

Muscle formation is controlled by a number of key myogenic transcriptional regulators that govern stage-specific gene expression programs and act as terminal effectors of intracellular signaling pathways. To date, the role of phosphatases in the signaling cascades instructing muscle development remains poorly understood. Here, we show that a specific PP2A-B55δ holoenzyme is necessary for skeletal myogenesis. The primary role of PP2A-B55δ is to dephosphorylate histone deacetylase 4 (HDAC4) following myocyte differentiation and ensure repression of Myocyte enhancer factor 2D (MEF2D)-dependent gene expression programs during myogenic fusion. As a crucial HDAC4/MEF2D target gene that governs myocyte fusion, we identify *ArgBP2*, an upstream inhibitor of Abl, which itself is a repressor of CrkII signaling. Consequently, cells lacking PP2A-B55δ show upregulation of ArgBP2 and hyperactivation of CrkII downstream effectors, including Rac1 and FAK, precluding cytoskeletal and membrane rearrangements associated with myoblast fusion. Both in vitro and in zebrafish, loss-of-function of PP2A-B55δ severely impairs fusion of myocytes and formation of multinucleated muscle fibers, without affecting myoblast differentiation. Taken together, our results establish PP2A-B55δ as the first protein phosphatase to be involved in myoblast fusion and suggest that reversible phosphorylation of HDAC4 may coordinate differentiation and fusion events during myogenesis.

## Introduction

Skeletal myogenesis, the process by which contractile muscle fibers are formed begins with specification of precursor cells towards the myoblastic lineage, followed by differentiation into postmitotic myocytes. Differentiated myoblasts then fuse to either one another to form new multinucleated myofibers or with an existing myofiber to support muscle growth. Surprisingly, there is still a relative lack of knowledge about the cellular and molecular mechanisms underlying myoblast fusion, especially when compared to the events that precede it^1^.

Developmental progression in the skeletal muscle lineage is orchestrated by a group of basic helix-loop-helix (bHLH) transcription factors collectively referred to as myogenic regulatory factors (MRFs). The ability of MRFs to drive the myogenic program relies on their collaboration with non muscle-specific factors, such as MEF2 family members and chromatin-modifying enzymes^2^. Whereas MEF2 has acquired a central role in multiple aspects of the skeletal muscle differentiation program^3^, its potential function during myoblast fusion remains essentially unaddressed. In myoblasts, MEF2 transcriptional activity is negatively regulated by its interaction with class IIa histone deacetylases (HDACs), including HDAC4, −5, −7, and −9^4^. Association with class IIa HDACs decreases MEF2 DNA-binding and leads to repression of MEF2-mediated transcription. Consequently, class IIa HDACs are potent inhibitors of skeletal muscle differentiation^4–8^. During myoblast differentiation, the inhibitory action of class IIa HDACs is overcome by their phosphorylation on conserved serine residues by myogenic kinases^9^. Phosphorylation leads to the disruption of MEF2-HDAC complexes, allowing MEF2 to activate the expression of pro-myogenic genes^10^. As exemplified for class IIa HDACs, protein phosphorylation is a key signaling event in the control of myogenic gene expression programs. Dozens of kinases have been reported to influence various stages during myogenesis^11^. In contrast, there is only very limited information about the role of the antagonistic phosphatases during skeletal muscle development, especially with respect to fusion.

Protein phosphatase 2A (PP2A) is a major serine/threonine phosphatase in adult and embryonic cells and is involved in a myriad of cellular signaling pathways^12–14^. PP2A is a trimeric complex built up by a core dimer consisting of a scaffolding A and catalytic C subunits associated with a third B-type regulatory subunit. There are 15 different B-type subunits, which are classified into four distinct families: PR55/B or B55, PR61B’ or B56, PR72B”, and STRN/PR93/PR110/B”. The B-type subunit is a crucial determinant in holoenzyme assembly, defining substrate specificity and providing the basis for the multiple physiological functions of PP2A^15^. One of the biggest challenges in the field remains the identification of the biological functions associated with each PP2A holoenzyme on the basis of its B-type subunit.

Here, we explored the role of PP2A in myogenesis and found that Bδ−containing PP2A holoenzymes control HDAC4-mediated repression on MEF2 transcriptional activity during myocyte fusion. Surprisingly, while inactivation of PP2A-Bδ severely affected myotube formation in vitro and in vivo, it had no effect on myoblast differentiation. In complement to the current model of myogenesis regulation by HDAC4 that is exclusively centered on myogenic kinases, our results highlight a more subtle mechanism in which temporal control of HDAC4 phosphorylation is also achieved by antagonizing phosphatases and participates in the regulation of both myoblast differentiation and myocyte fusion.

## Materials and methods

### Cell culture and reagents

C2C12 cells were purchased from ATCC (CRL-1772^TM^). Cells were routinely cultivated in Growth Medium (GM; Dulbecco’s Modified Eagle Medium (DMEM) with 20% Fetal Bovine Serum (FBS) and supplemented with penicillin/streptomycin). For differentiation experiments, cells were grown up to 70–80% confluence (Day 0) in GM and switched into Differentiation Medium (DM; DMEM with 2% horse serum supplemented with penicillin/streptomycin) for the indicated time. Okadaic acid was purchased from Merck (459620). PP2Acα and PP2Acβ siRNAs were purchased from Santa-Cruz Biotechnology (sc-36302 and sc-43540, respectively). Plasmids encoding rat Ba and Bd homologs were kind gifts from Dr D. Virshup (Duke-NUS medical school). For Zebrafish injection, both ORF were subcloned into the pCS2 + vector.

C2C12 stably expressing shRNAs against B55δ (Sigma-Aldrich TRCN0000080900: CCCACATCAGTGCAATGTATT, TRCN0000080902: CGGTTCAGACAGTGCCATTAT) were generated as follows. In brief, Lenti-X 293 T cells (Clontech, Belgium, 632180) were co-transfected with a pSPAX2 (Addgene, Plasmid #12260), a VSV-G encoding vector^16^ and with pLKO shRNA plasmids targeting B55δ or containing non target sequence (Sigma, SHC005 and SHC002). Forty-eight and 72 h post-transfection, viral supernatants were collected, filtrated, and concentrated ~100 fold by ultracentrifugation. C2C12 cells were transduced three times with concentrated viral supernatant and transduced cells were selected with 0.5 µg/mL puromycin. Finally, the supernatants from selected polyclonal C2C12 were checked for the absence of RCL (Replication Competent Lentivirus) before used.

### Immunochemistry and microscopy

C2C12 cells were seeded at 70–80% confluence onto cover-slips at day 0 and, at the indicated time points, fixed in 4% paraformaldehyde, permeabilized in 0.1% Triton X-100 for 10 min, and blocked in 3% Bovine Serum Albumin (BSA) for 1 h. Cells were then incubated in indicated primary antibody (at appropriate dilution) for 1 h, washed three times in phosphate-buffered saline (PBS) and then further incubated in the appropriated secondary fluorescent antibody (Alexa488, 568 and/or 633, at 1/500 dilution). Nuclei were labeled by a 10 min incubation in Hoescht 33342. Cover-slips were mounted and analyzed using a Nikon A1R or Leica SP5 confocal microscope.

Zebrafish embryos were fixed using 4% paraformaldehyde overnight at 4 °C, washed three times with PBS-0.3% Triton (PBS-0.3%T) and then were permeabilized in PBS-1% Triton for 15 min at room temperature (RT). Embryos were blocked 2 h at RT in PBS-0.1%T-4% BSA, subsequently incubated in indicated primary antibodies or phalloïdin-Alexa488 overnight at 4 °C and then washed six times in PBS-0.3%T. Embryos were then incubated in secondary fluorescent antibody (Alexa fluor 488, 568, and/or 633) at a 1/500 dilution overnight at 4 °C and finally washed six times in PBS-0.3%T. Nuclei were labeled by a 30-min incubation in Hoescht 33342 (1/20,000). Embryos were mounted and imaged using a Nikon A1R, a Leica SP5, or a Zeiss LSM 710 (objective: Plan-Apochromat × 20/0.8 M27) confocal microscope.

For live imaging, cells were transfected at 70–80% confluence with Utrophin-GFP using Lipofectamine 2000 (Thermo Fisher Scientific®). Proliferating (day 0) or differentiating (day 2) myoblasts transfected cells were reseeded into a 2-well Lab-Tek® II Chamber Slide™ and imaged using an AR1 Nikon confocal microscope for 4 h.

### Zebrafish and morpholino injection

AB Zebrafish lines were maintained according to EU regulations on laboratory animals. The animal welfare committee of the University of Liège approved all animal experiments. Knockdown experiments were performed by injecting embryos at the one- to two-cell stage with 3 ng of single morpholino or 3–8 ng of combined morpholinos. Stable antisense morpholino nucleotides were obtained from GeneTools: PP2A-Bδ (Mo#1: ACCAACCCCTGCCATCATCGCCTGT at 3 ng/nl or MO#2: AAGTCTTTGAGTTGCATTACCTCCT at 10 ng/nl) and ArgPB2 (Mo#A: GTTGGCTCCATGCCTGATACCTGCA at 5 ng/nl together with MO#B: GCCTCCACAAATCTAATAGACAAAC at 1 ng/nl). Bright-field images were taken from live embryos at 48 hpf using a stereomicroscope, whereas confocal pictures were taken on fixed embryos using a Nikon A1R or Leica SP5 confocal microscope.

### GTPase pull-down activity assay

C2C12 cells were chilled on ice and harvested at the indicated time points. Cells were then lysed in ice-cold lysis buffer (1% Triton X-100, 25 mM HEPES pH 7.3, 150 mM NaCl, 4% glycerol, proteases, and phosphatases inhibitor). Lysates were centrifuged for 2 min at 13,000×*g* and supernatants were flash-frozen in liquid nitrogen and stored at −80 °C. For pull-down assays, supernatants were incubated 30 min with 30 μg of GST-PBD protein for Cdc42 and Rac1 activity measurement or with 30 μg of GST-PBD protein for RhoA activity measurement. GST-pull-downs were washed four times in lysis buffer and analyzed by western Blotting using the appropriate antibody. Total GTPase levels were estimated by western blotting of inputs^17^.

### Fusion and differentiation index

Fusion index was defined as the number of nuclei inside MHC + cells containing at least two nuclei divided by the total number of nuclei within MHC + cells. Differentiation index was defined as the number of nuclei inside MHC + cells divided by the total number of nuclei.

### Cell transfection

C2C12 cells were transfected using Lipofectamine 2000 with the indicated plasmids, according to the manufacturer’s instructions. Transfection medium was removed after 24 h and replaced with DM or GM, depending on the assay. SiRNA were transfected using ProFection® Mammalian Transfection System (Promega) according to the manufacturer’s recommendations. In brief, cells were transfected at 70–80% confluence in 24-well plates with 16 pmol of the indicated siRNA. The next day, medium was replaced by GM or DM. SiRNA against PP2A B and B′ regulatory subunits were purchased from Sigma-Aldrich.

### Viability assay

Cell viability was assessed at the indicated time point with CellTiter 96® AQ_ueous_ One Solution MTS assay (Promega) according to the manufacturer’s instructions.

### Morphology analysis

Cells were grown up to 70–80% confluence in 24-well plates in GM, harvested and reseeded in GM or DM at low density on cover-slips. At indicated time points, cells were labeled with CellMask deep red (LifeTechnologies, 5 µg/mL for 20 min at 37 °C), fixed in 4% paraformaldehyde and mounted for confocal microscopy analysis. In rescue experiments, C2C12 cells were incubated with Rac1 inhbitor NSC23766 (Sigma Aldrich) (50 µM) 24 h before analysis. Major and minor axis ratio was calculated using the ImageJ software.

### ChIP experiments

HighCell ChIP kit (Diagenode) was used according to the manufacturer’s recommendations. In brief, 10^7^ cells were harvested at the indicated time points and fixed with formaldehyde. After addition of glycine, chromatin was fractionated using a Bioruptor (Diagenode) (two runs of 10 cycles, 30 s on-30s off). Indicated antibodies were added in cell lysate at appropriate concentrations and incubated overnight à 4 °C. After washing, DNA was purified and submitted to qPCR analysis with primers mapping to the ArgPB2 promoter. Amplification of the *Gapdh* promoter was used as negative control. Data were processed based on the percentage input method according to the kit manual.

### RNA extraction and PCR amplification

RNA was extracted using the nucleospin RNA kit (Macherey Nagel) according to the manufacturer’s protocol. RNA purity and concentration were assessed by spectrophotometry analysis (Nanodrop, Thermo Scientific). Reverse transcription reactions were done using the RevertAid H Minus First Strand cDNA Synthesis Kit (Fermentas) with random hexamer primers. The cDNA was then submitted to quantitative real-time PCR (qPCR) using LightCycler®480 SYBR Green I Master (Roche Applied Science) on a LightCycler®480 apparatus ((Roche Applied Science).

### RNA-seq analysis

RNA was extracted using the nucleospin RNA kit (Macherey Nagel) according to the manufacturer’s protocol. RNA purity and concentration were assessed by spectrophotometry analysis (Nanodrop, Thermo Scientific). RNA-Seq libraries were prepared according to the TruSeq stranded mRNA protocol (Illumina) and 75-bp single-end reads produced on a NextSeq500 instrument. Reads were mapped to the Mus musculus transcriptome (Ensembl cDNA release 96) and quantified using Salmon v0.8.2^18^. Read counts were summed to the gene level using tximport^19^ and differential expression was assessed using DESeq2^20^. Clustering was performed and heatmaps of log2-transformed fold change values were generated using the R package heatmap v1.0.8. Gene Ontology (GO) term enrichment analysis was performed using DAVID 6.8^21^. MEF2 targets in myoblasts were previously identified^22^.

### Cell cycle

C2C12 cells were washed with PBS, fixed in 0.5% paraformaldehyde at 4 °C for 20 min and permeabilized in 70% ethanol for 30 min at 4 °C. After PBS washing, RNAse A (final concentration: 0.5 mg/mL) and propidium iodide (final concentration: 100 µg/mL) were added. Cells were analyzed using a FACScalibur cytometer (Becton Dickinson) and data were analyzed using ModFit LT (Very Software House).

### Statistical analysis

Unless stated otherwise, graph values are presented as means±standard deviations, calculated on at least three independent experiments. Depending on the results of normality tests, statistical significance was determined using two-tailed Student’s *t*-test, one-way ANOVA test with a Bonferroni’s or Tukey’s adjustment, one-sample *t*-test, Mann–Witney’s test or Kruskal–Wallis ‘ test with a Dunn’s adjustment. Western blots quantifications were performed on images from at least two separate experiments using ImageJ software, with representative images shown.

## Results

### PP2A activity is necessary for myogenesis

**Fig. 1:**
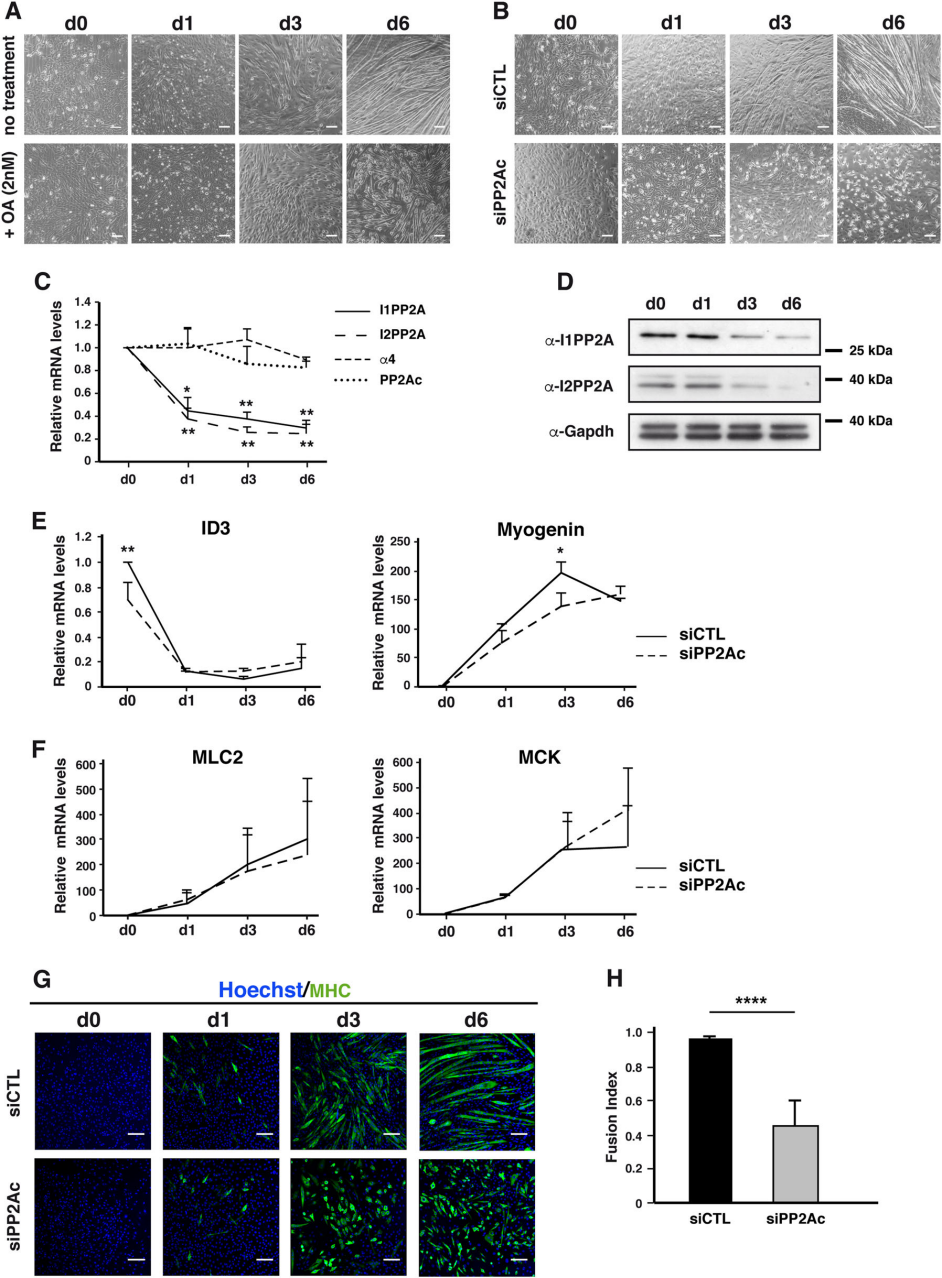
PP2A is required for myogenesis of C2C12 myoblasts. **a** Phase contrast images of C2C12 myoblasts treated (+OA) or not (no treatment) with 2 nM of okadaic acid. Pictures were taken at the indicated time points during differentiation and are representative of four experiments. **b** Phase contrast images of differentiating C2C12 myoblasts transfected with a control (siCTL) or a PP2Ac (siPP2Ac) siRNA. Pictures were taken at the indicated time points and are representative of three experiments. **c** RT-qPCR analysis of the indicated mRNA at day 0 (d0), day 1 (d1), day 3 (d3), and day 6 (d6) during differentiation of wild-type C2C12 myoblasts (*n* = 3, one-way anova, with Tukey’s post-hoc test, **P *<* 0.01, *P *<* 0.05). **d** Western blot analysis of I1PP2A and I2PP2A protein levels during differentiation of wild-type C2C12 cells, representative of two experiments. **e**, **f** RT-qPCR analysis **e** early (ID3 and Myogenin, *n* = 3) and **f** terminal (MLC2, *n* = 3 and MCK, *n* = 3) differentiation markers in differentiating C2C12 myoblasts transfected with a control (siCTL) or a PP2Ac (siPP2Ac) siRNA. Two-way anova, with Bonferroni correction, **P *<* 0.01, *P *<* 0.05. **g** Confocal imaging of differentiating C2C12 myoblasts transfected with a control (siCTL) or a PP2Ac (siPP2Ac) siRNA and stained for MHC expression (green), representative of three experiments. Nuclei (Hoechst) are in blue. **h** Fusion index at 6 days during differentiation (see methods for details) of C2C12 cells transfected with a control (siCTL) or a PP2Ac (siPP2Ac) siRNA (*n* = 4, unpaired *t*-test, two-tailed, ****P *<* 0.0001). Scale bars are 100 µm. Values are mean ± SD

To explore a potential role for PP2A in myogenesis, C2C12 myoblasts were cultured in differentiation medium (DM) with and without low concentration (2 nM) of the PP2A inhibitor Okadaic Acid (OA) (IC50 = 0.1 nM)^23^. After 6 days in DM, control C2C12 cells showed extensive formation of multinucleated myotubes (Fig. 1a). In contrast, the OA-treated C2C12 were dramatically impaired in their ability to form myotubes. As an alternative approach, we depleted PP2A activity from C2C12 cells by knocking down its catalytic subunit (PP2Ac) using a specific siRNA (Supplementary Fig. 1A, B). Knockdown of PP2Ac almost completely abolished the capacity of C2C12 to form multinucleated myotubes (Fig. 1b). Messenger RNA levels of PP2Ac and α4, an atypical subunit that modulates PP2Ac stability remained stable during the myogenic process^24^ (Fig. 1c). However, expression levels of I1PP2A and I2PP2A, two known endogenous inhibitors of cellular PP2A activity^25^ were dramatically reduced as myoblasts progressed into the myogenic program (Fig. 1c, d). Altogether, these observations indicate that minimal levels of PP2A activity are required for myotube formation.

### PP2A is involved in fusion of myocytes into myotubes

Depletion of PP2Ac had no significant effect on C2C12 ability to withdrew from the cell cycle (Supplementary Fig. 1C, D) or their viability (Supplementary Fig. 1E). In addition, we found no significant differences in the mRNA levels of *inhibitor of differentiation 3 (ID3)* and *myogenin*, two effectors involved in the initiation of differentiation^26,27^ between siCTL and siPP2Ac-transfected cells (Fig. 1e and Supplementary Fig. 1F), suggesting that PP2A is not necessary for early differentiation. The terminal differentiation markers muscle creatine kinase (MCK) and myosin light chain 2 (MLC2) were induced to the same levels in PP2Ac-depleted and control cells (Fig. 1f, Supplementary Fig. 1F). Expression levels of myosin heavy chain (MHC), a late differentiation marker were also comparable in siCTL- and siPP2Ac-treated myoblasts (Fig. 1g). In addition, siCTL and siPP2Ac myoblasts had similar differentiation index (Supplementary Fig. 1G). Altogether, these results showed that lack of PP2Ac had no impact on myogenic differentiation. Although they express MHC, we noticed that PP2Ac-KD myocytes showed noticeable morphological differences compared to control cells. At day 3, the alignment of PP2Ac-depleted cells was reduced and cells appeared less elongated (Fig. 1g). In addition, PP2A-deficient cells formed dramatically less multinucleated myotubes and when present, these myotubes were notably smaller and contained fewer nuclei. Analysis of the fusion index confirmed these observations and indicated that PP2A is important for the late stage of myogenesis, when differentiated myoblasts start to fuse to each other (Fig. 1h).

### PP2A-Bδ holoenzyme specifically controls myocyte fusion in vitro

**Fig. 2:**
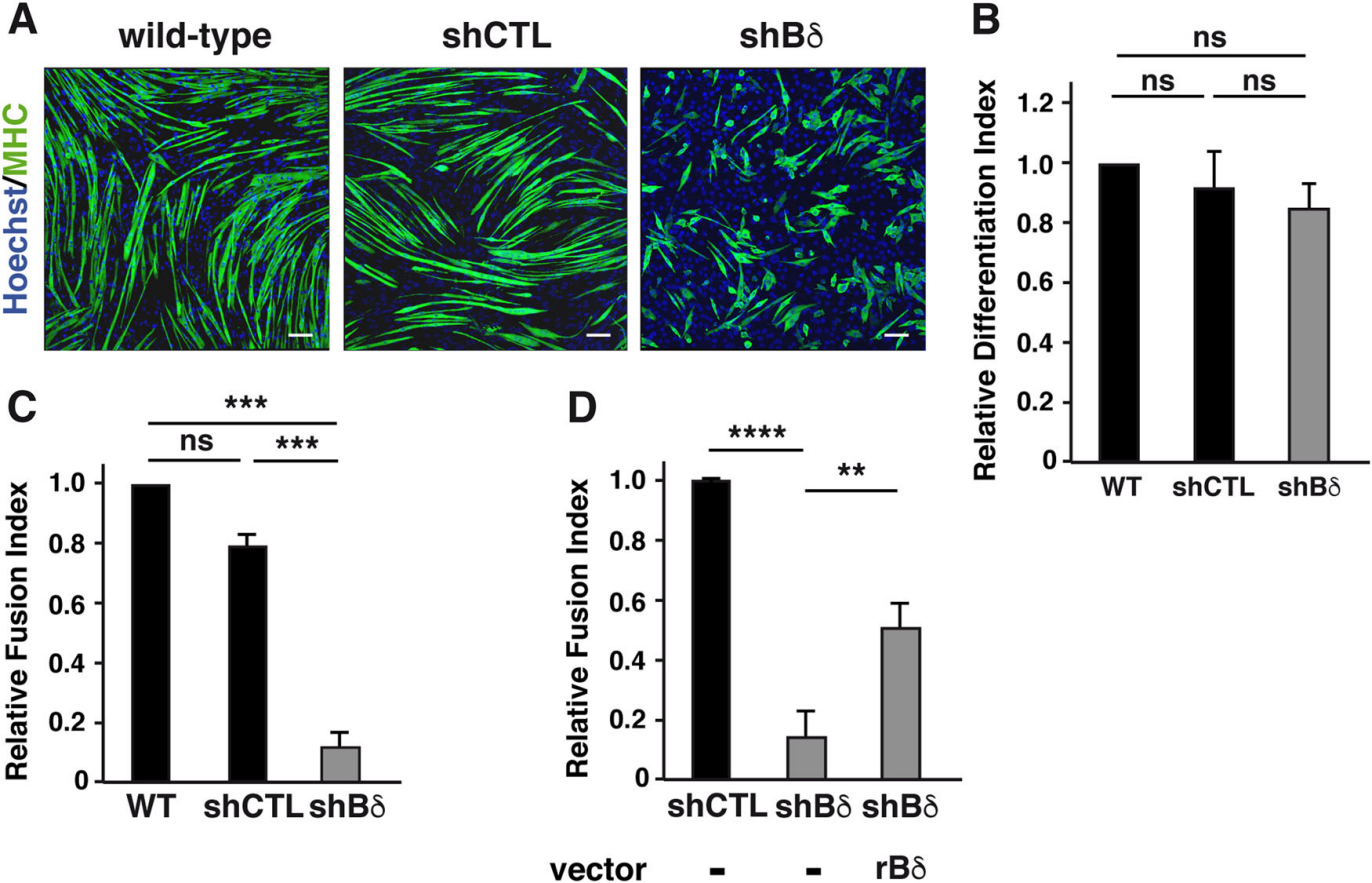
PP2A-Bδ holoenzyme specifically controls myocyte fusion in vitro. **a** Confocal images of MHC (green) expression in wild-type C2C12 myoblasts or C2C12 myoblasts stably expressing a control (shCTR) shRNA or two shRNA against B55δ (shBδ), at day 6 during differentiation. Nuclei are stained with Hoechst (blue). Scale bars are 100 µm. Images are representative of three experiments. **b**, **c** Differentiation (**b**) and fusion (**c**) index (see methods for details) of wild-type C2C12 myoblasts or C2C12 myoblasts stably expressing a control shRNA (shCTR) or two shRNA against B55δ (shBδ). *N* = 3, one-way anova, with Tukey’s post hoc test. ***P *<* 0.001, ns: not significant. **d** Fusion index of control (shCTL) and Bδ-knocked down (shBδ) C2C12 myoblasts transfected with an empty vector (−) or a vector coding for rat PP2A-Bδ. *N* = 4, one-way anova with Tukey’s post-test, **P *<* 0.01, ****P *<* 0.0001

To identify the specific PP2A holoenzyme driving fusion of myocytes, we first examined the expression of PP2A B and B′ regulatory subunits in C2C12 cells. Except for Bγ, we detected all B (Bα, -β and -δ) and B′ subunits (B′α, -β, -γ, -δ, and -ε) by RT-qPCR (data not shown). Using a series of siRNAs we reduced the expression of each B and B′ subunits (Supplementary Fig. 2A) and assessed the effects on the fusion index of differentiating myocytes. Although minor, the most significant effect was obtained after knocking down the Bδ subunit (Supplementary Fig. 2B). Suspecting that dilution of the siRNA efficiency over the 6 days period might prevent pronounced defects, we generated a polygenic C2C12 myoblast line in which expression of Bδ was significantly reduced by stably expressing two shRNA (Supplementary Fig. 2C, D). To confirm the specificity of the shRNA we verified that expression of Bα and Bγ, the other B subfamily members was not affected in the Bδ-depleted cells (Supplementary Fig. 2E). When switched to DM, Bδ−KD myoblasts withdrew from the cell cycle as efficiently as wild-type or control cells (Supplementary Fig. 2F). After 6 days of differentiation, many MHC-positive cells were observed in the PP2A-Bδ-depleted culture (Fig. 2a). Indeed, the differentiation index was similar in shBδ-expressing and control cell lines (Fig. 2b), indicating that lack of this particular PP2A holoenzyme had no significant effect on differentiation. In contrast, PP2A-Bδ-depleted myocytes appeared unable to elongate and form multinucleated myotubes (Fig. 2a–c). Importantly, fusion defects were also observed in myoblasts transduced with each Bδ shRNA independently (Supplementary Fig. 2G). In addition, expression of a shRNA-insensitive rat Bδ ORF significantly restored the ability of myocytes to fuse into myotubes (Fig. 2d). Altogether, these findings support the idea that PP2A-Bδ is specifically involved in the regulation of myocyte fusion in vitro.

### PP2A-Bδ holoenzyme is necessary for myofiber formation in vivo

**Fig. 3:**
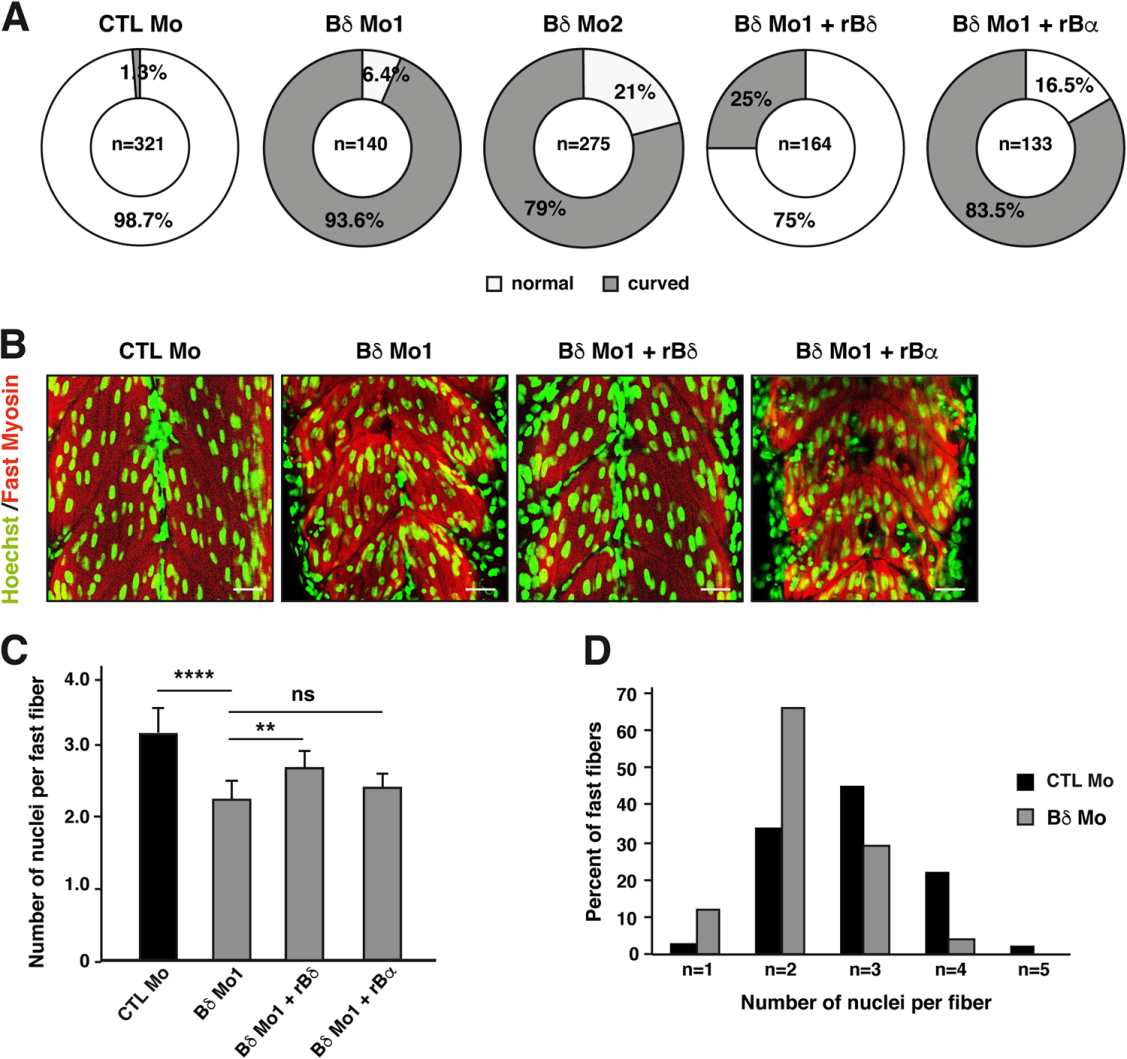
PP2A-Bδ holoenzyme is necessary for myofiber formation *in vivo*. **a**, **b** Quantification of the curved trunk phenotype (**a**) and **b** representative confocal imaging in 48-hpf zebrafish embryos injected with a control morpholino (CTL Mo), with an ATG-blocking morpholino alone (Bδ Mo1) or together with a rat Bδ (Bδ Mo1 + rBδ) or rat Bα mRNA (Bδ Mo1 + rBα) or an alternative splice-blocking morpholino (Bδ Mo2) against PP2A-Bδ. Nuclei (Hoechst) and fast skeletal myosin are shown respectively in green and red. Scale bars are 25 µm. **c** Quantification of the number of nuclei in fast skeletal myofibers in 48-hpf control embryos (CTL Mo, *n* = 16) or in embryos injected with the PP2A-Bδ Mo alone (Bδ Mo, *n* = 20) or together with a rat Bδ (Bδ Mo + rBδ, *n* = 7) or rat Bα mRNA (Bδ Mo1 + rBα, *n* = 9). Values are mean ± SD, unpaired *t*-test, two-tailed, ****P *<* 0.0001. **d** Proportion of fibers with the indicated number of nuclei in control (CTL Mo, *n* = 11) or PP2A-Bδ (Bδ Mo, *n* = 14) morphant embryos

To validate the role of PP2A-Bδ in myogenesis in vivo, we turned to the zebrafish model. A single Bδ ortholog was identified in the zebrafish genome (ENSDARG00000102009), against which we designed an ATG-blocking antisens morpholino (BδMo1) (Supplementary Fig. 3A). Even at low doses of morpholino (3 ng/embryo), a significant proportion of Bδ morphants exhibited a curved tail and showed mobility defects (Supplementary Fig. 3B and Fig. 3a), suggesting myoblast fusion defects^28^. Importantly, co-injection of a rat Bδ mRNA fully restored normal body straightness in PP2A-Bδ morphants, while injection of the rat Bα mRNA did not. In addition, an alternative splice-blocking morpholino (BδMo2) led to similar defects (Fig. 3a).

In Bδ^KD^ embryos, somites had the classic chevron shape and we found no statistical differences in the somite angles when compared to control embryos (Supplementary Fig. 3C). Slow fibers were observed laterally in Bδ morphants and showed regular spacing and constant length, despite a slight disorganization and occasional gaps (Supplementary Fig. 3D). In addition, the numbers of slow fibers per somite were comparable in control and Bδ morphants (Supplementary Fig. 3E). Together, these findings indicated that Bδ knockdown did not affect slow fiber formation. In contrast, the fast myotome was dramatically altered in Bδ morphants, which exhibited twisted fast myofibers with erratic nuclei organization (Fig. 3b). Although fast-twitch fibers of Bδ morphants displayed regular sarcomeric cross striation, a feature of mature myofibers (Supplementary Fig. 3F), they had significantly less nuclei, indicating fusion defects (Fig. 3c). Indeed, the proportion of long multinucleated fibers was dramatically smaller in Bδ morphants with the vast majority (77%) of myotubes containing only one or 2 nuclei (Fig. 3d). Importantly, a similar phenotype was also observed using the splice-blocking morpholino (Supplementary Fig. 3G,H). In addition, co-injection of the rat Bδ mRNA partially reverted fusion defects in Bδ morphants, while injection of the related Bα was ineffective (Fig. 3b, c). Collectively, these findings strongly support the idea that PP2A-Bδ holoenzyme is specifically required for myoblast fusion in vivo.

### PP2A-Bδ holoenzyme is required for fusion-associated cytoskeleton morphogenesis

**Fig. 4:**
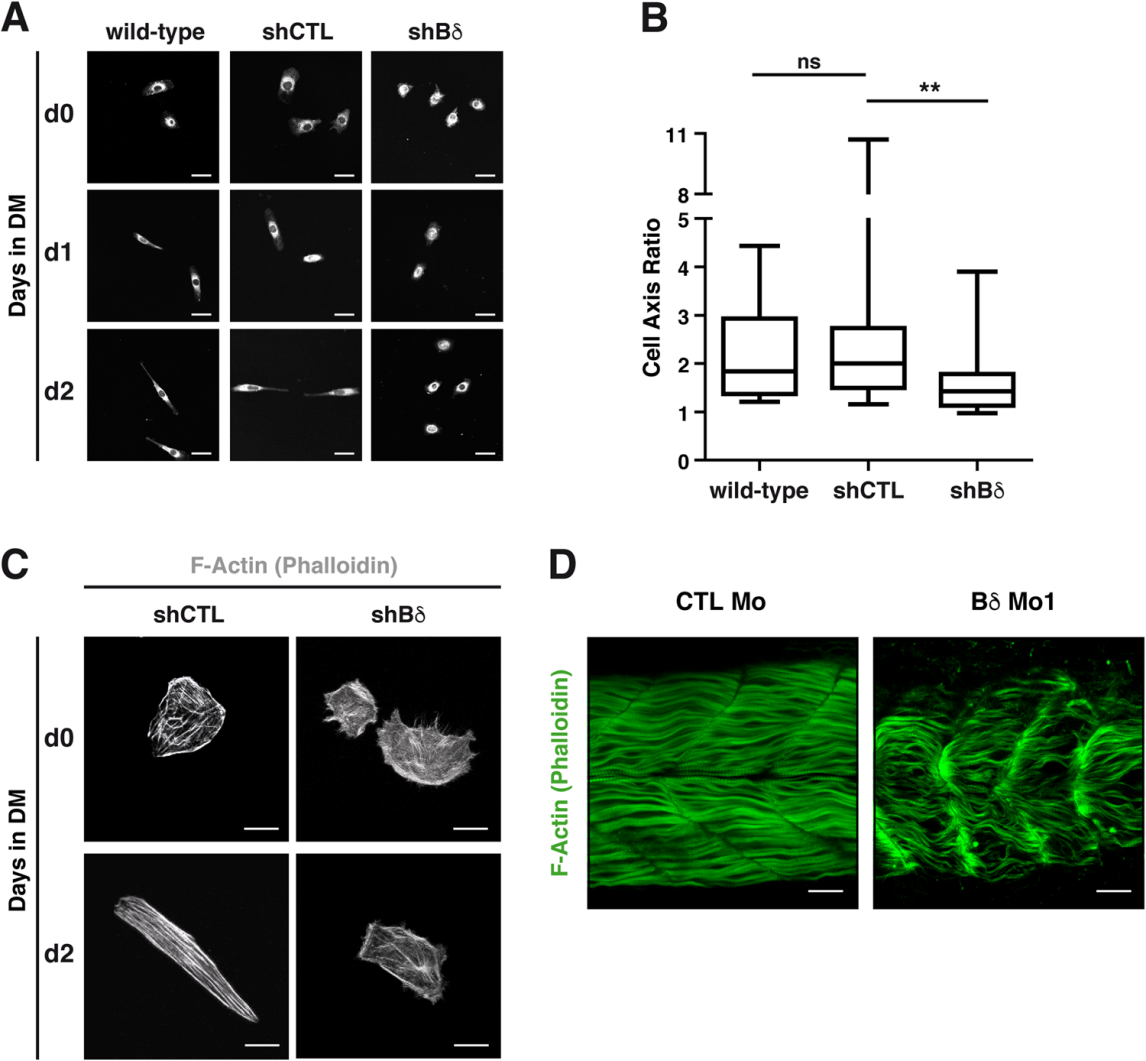
PP2A-Bδ is involved in fusion-associated cytoskeleton morphogenesis. **a** Representative (*n* = 2) confocal images of morphology of wild-type (WT), control (shCTL), and Bδ-knocked down (shBδ) C2C12 myoblasts at the indicated time points during the differentiation process. Cells were stained with CellMask (white). Scale bars are 25 µm. **b** Major/minor cell axis ratio in wild-type (*n* = 21), control (shCTL, *n* = 29), and Bδ-knocked down (shBδ, *n* = 27) C2C12 myoblasts at day 2 during the differentiation process. Kruskal–Wallis with Dunn’s correction, *P *<* 0.05, ^**^P *<* 0.01. ns: not significant. **c** Confocal analysis of F-actin (phalloidin, gray) in control (shCTL) and Bδ-knocked down (shBδ) C2C12 myoblasts grown in GM (d0) or 2 days in DM (d2). Representative images from two experiments are shown. Scale bars are 10 µm. **d** Representative (*n* = 3 experiments) confocal imaging of F-actin (phalloidin, green) in 48-hpf control (CTL Mo) or PP2A-Bδ (Bδ Mo1) morphant embryos. Scale bars are 25 µm

After 2 days in DM, control myoblasts went from their characteristic fibroblast-like appearance to an elongated, spindle-shaped morphology (Fig. 4a)^29^. In contrast, Bδ^KD^ cells mostly retained their myoblastic appearance when cultured in DM. The measure of the cell axis ratio (major axis/minor axis) confirmed that after 2 days in DM Bδ^KD^ cells were significantly less elongated than control cells (Fig. 4b). Interestingly, control and Bδ^KD^ cells had a similar cell axis ratio when cultured in GM, indicating that PP2A-Bδ is not involved in maintaining the morphology of proliferating myoblasts (Supplementary Fig. 4A). Adoption of bipolar shape and fusion are driven by reorganization of the actin cytoskeleton^30^. Thus, we examined actin organization in Bδ-KD myoblasts. After 2 days in DM, F-actin was mostly concentrated at the periphery of bipolar control myoblasts (Fig. 4c). However, a number of short longitudinal actin bundles were also visible, suggesting increasing cell contractility as myoblasts proceed through differentiation^29^. In contrast, cellular tension was severely reduced in Bδ^KD^ myoblasts. Although some actin bundles had formed after 2 days in DM, they were consistently thinner than in control cells and highly disorganized. Of note, tubulin dynamics was not affected in Bδ-depleted myoblasts, as assessed by tubulin acetylation (Supplementary Fig. 4B). Interestingly, actin polymerization defects were also observed in vivo as Bδ morphants exhibited thinner and less organized actin fibers in fast-twitch muscles (Fig. 4d).

### PP2A-Bδ holoenzyme regulates cytoskeleton dynamics via the RhoGTPase Rac1

**Fig. 5:**
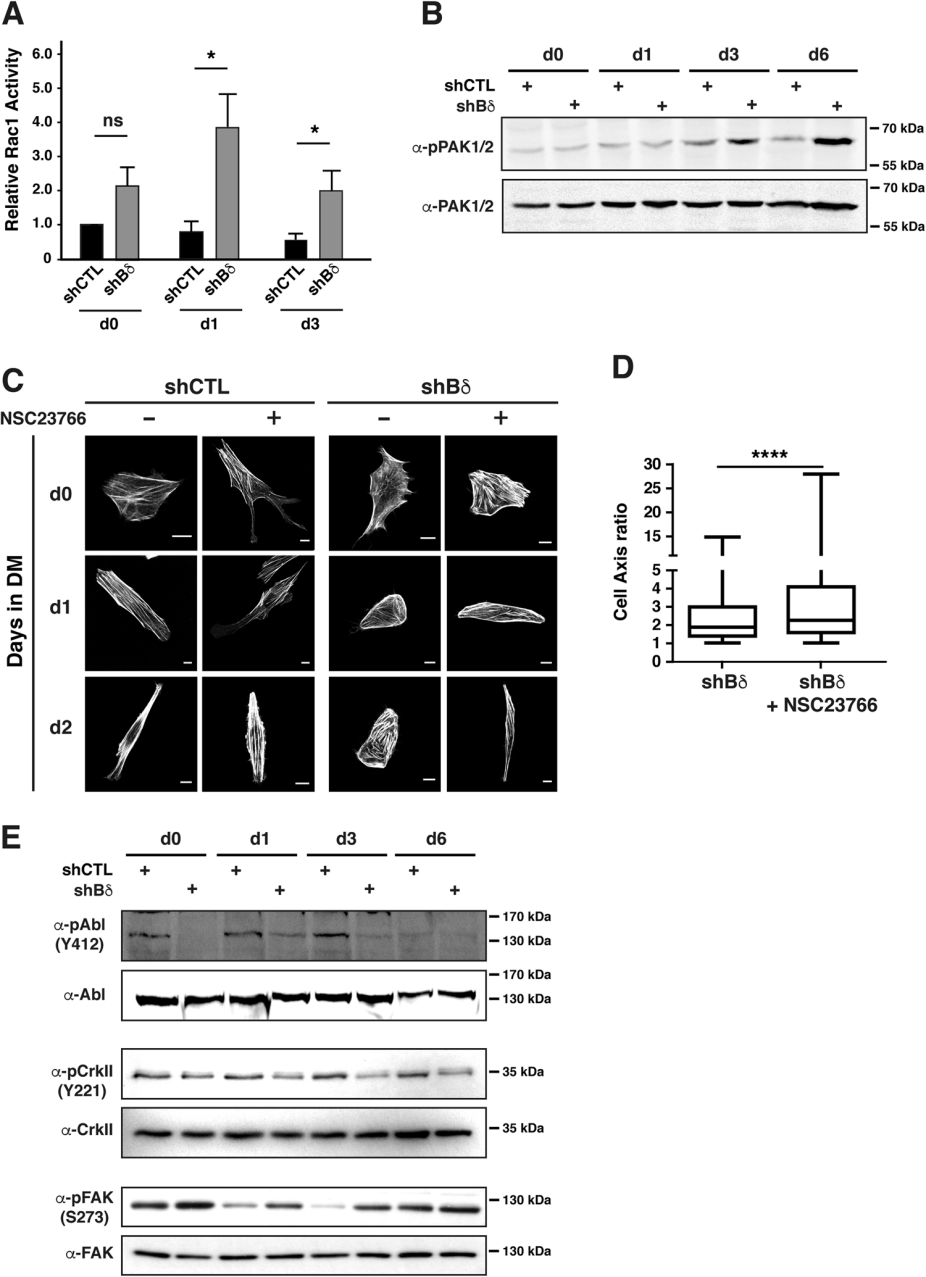
PP2A-Bδ holoenzyme regulates cytoskeleton dynamics via the RhoGTPase Rac1. **a** Rac1 activity was measured by GST pull-down assay in control (shCTL) and Bδ-knocked down (shBδ) C2C12 myoblasts at the indicated time points during the differentiation process. Values are mean ± SD from at least three experiments. Unpaired *t*-test, two-tailed, *P *<* 0.05. **b** Western blot analysis of phospho-Pak1,2 (α-pPAK1,2) and total Pak 1,2 (α-PAK1,2) in control (shCTL) and Bδ-knocked down (shBδ) C2C12 myoblasts at the indicated time points during the differentiation process. Images are representative of two independent experiments. **c** Confocal analysis of F-actin (phalloidin, gray) in control (shCTL) and Bδ-knocked down (shBδ) C2C12 myoblasts treated ( + ) or not (−) with a Rac1 inhibitor (NSC23766). Imaging was done at the indicated time points during the differentiation process. Representative cells from two independent experiments are shown. Scale bars are 10 µm. **d** Major/minor cell axis ratio in control Bδ-KD (shBδ) C2C12 myoblasts treated or not with the NSC23766 Rac1 inhibitor. Analysis was performed at day 2 during the differentiation process. Cells from 2 independent experiments for each condition were analyzed (*n* = 570 for shBδ and *n* = 585 for shBδ + Rac inhibitor). Mann–Witney’s test ****P *<* 0.0001. **e** Analysis of Abl, CrkII, and FAK activation by western blot in control (shCTL) and Bδ-KD (shBδ) C2C12 myoblasts during differentiation using antibodies against the phosphorylated forms of their activation sites, i.e., Y412 (α-pAbl (Y412)), (α-pCrkII (Y221)), and (α-pFAK (S273)), respectively. In total, Abl, CrkII, and FAK were used as loading controls. Images are representative of at least two experiments

Cellular cytoskeleton organization is under the control of the Rho family of small guanosine triphosphatases (GTPases) and previous studies have identified RhoA, Rac1, and Cdc42 as important regulators of myogenesis^1^. We observed no difference in RhoA activity levels in Bδ–KD and control C2C12 cells (Supplementary Fig. 5A)^31^. Although Cdc42 activity was slightly higher in proliferating Bδ^KD^ myoblasts, this difference decreased as cells progress through differentiation (Supplementary Fig. 5B). Rac1 activity levels were higher in Bδ-depleted cells, especially between day 1 and 3, when fusion occurs (Fig. 5a). Interestingly, previous work has demonstrated that Rac1 prevents actin polymerization into stress-fibers and diminishes cell contractility, similar to what we observed in Bδ^KD^ myoblasts (Fig. 4c). Although the exact mechanism is still unclear, the function of Rac1 on actin polymerization seems to be mediated by downstream effector p21-activated kinases (PAKs)^32^. In agreement with increased Rac1 activity and the absence of stress fiber-like actin bundles, we found significant hyperactivation of PAK1/2 in Bδ^KD^ cells (Fig. 5b). While having a negative effect on stress fiber assembly, the Rac1-PAK axis promotes polymerization of cortical actin, assembly of lamellipodial small focal adhesions (FAs), and membrane protrusions^33^. In control cells cultured 2 days in DM, membrane remodeling was mostly restricted to each tip of the bipolar myoblasts (Supplementary video 1). In contrast, Bδ-depleted myoblasts exhibited large regions of lamellipodia undergoing expansion and retraction cycles (Supplementary video 2), a membrane behavior compatible with activation of the Rac1-PAK axis^34^. When Bδ-KD myoblasts were allowed to differentiate in the presence of sub-optimal concentrations of NSC23766, a specific Rac1 inhibitor^35^, they elongated and adopted a spindle-like shape, similar to control differentiated myoblasts (Fig. 5c, d). Altogether, our data indicate that loss of PP2A-Bδ in myoblasts prevents proper regulation of Rac1-PAK signaling and interferes with downstream actin-based processes that are crucial for myoblasts fusion^35^.

### PP2A-Bδ holoenzyme regulates Rac1 activity via an Abl/CrkII pathway

CrkII is a key regulator of Rac1 during membrane ruffling and lamellipodial extensions^33^. Activation of CrkII allows formation of a p130Cas-Crk complex, which in turn recruits the Rac1 GEF Dock180/ELMO complex^36^. The scaffolding activity of CrkII towards p130Cas/Dock180/ELMO is inhibited by phosphorylation of its Tyr221 by the Abl tyrosine kinases^37^. Compared to controls, Bδ-depleted myoblasts exhibited lower activity of Abl and as expected, reduced phosphorylation of CrkII Tyr221 (Fig. 5e). Interestingly, decreased CrkII phosphorylation was especially visible between days 1 and 3 of differentiation, when fusion of myocytes starts (Fig. 5e). Besides Rac1, there is also evidence that CrkII can activate FAK-dependent signaling pathways, which has also been involved in myoblast fusion^1,38^. In agreement with their increased CrkII signaling, Bδ-KD cells exhibited significantly higher FAK activity (Fig. 5e)^39^. Altogether, these observations support the idea that lack of PP2A-Bδ is associated with inhibition of Abl and aberrant activation of CrkII-dependent signaling pathways. Among those, deregulation of Rac1 and FAK signaling would be expected to interfere with the cytoskeletal rearrangements, as they are important for myoblast fusion^35,40^.

### PP2A-Bδ holoenzyme represses MEF2D-dependent transcription through dephosphorylation of HDAC4

**Fig. 6:**
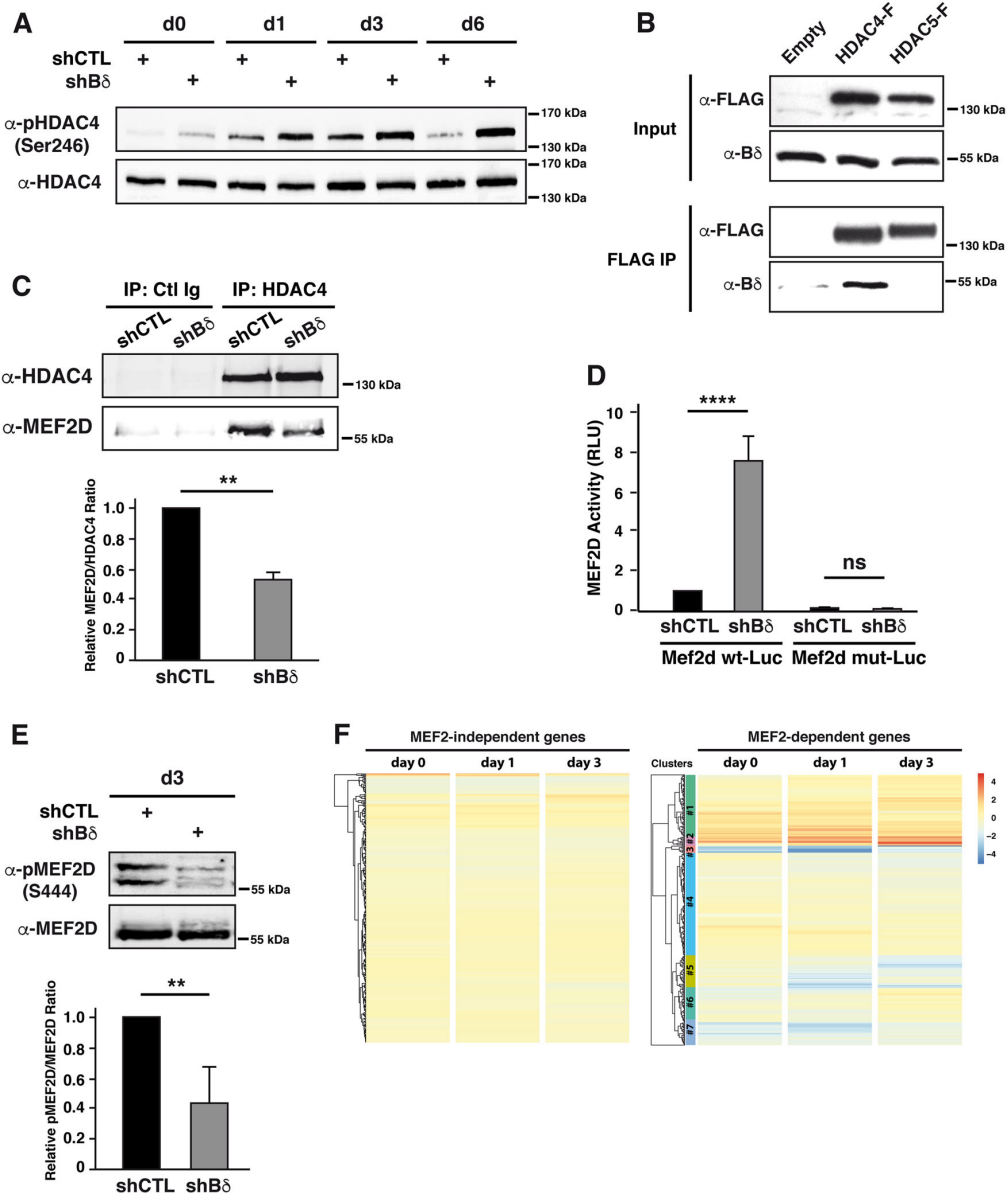
PP2A-Bδ holoenzyme represses MEF2D-dependent transcription through dephosphorylation of HDAC4. **a** Analysis of HDAC4 phosphorylation by western blot in control (shCTL) and Bδ-KD (shBδ) C2C12 myoblasts during differentiation using a phospho-specific antibody against its S246 (α-pHDAC4 (S246)). Total HDAC4 (α-HDAC4) was used as loading control. Representative of two independent experiments. **b** Western blot analysis of endogenous PP2A-Bδ levels co-immunoprecipitating with Flag-tagged HDAC4 or HDAC5 in proliferating C2C12 cells. Inputs or Flag immunoprecipitates (FLAG IP) were analyzed with anti-Flag (α-FLAG) or anti-PP2A-Bδ (α-Bδ) antibodies. Representative of two independent experiments. **c** Upper panel: co-immunoprecipitation between endogenous HDAC4 and MEF2D in control (shCTL) and Bδ-KD (shBδ) C2C12 myoblasts at day 3 of differentiation. Control (IP: Ctl Ig) or HDAC4 (IP: HDAC4) immunoprecipitates were analyzed by western blotting using anti-HDAC4 (α-HDAC4) and -MEF2D (α-MEF2D) antibodies. Lower panel: quantification of the amount of MEF2D in the HDAC4 immunoprecipites relative to the amount of HDAC4 calculated from three experiments as described in **c**, upper panel. One-sample *t*-test, **P *<* 0.01. **d** Analysis of MEF2 transcriptional activity in control (shCTL) and Bδ-KD (shBδ) C2C12 myoblasts at day 3. Cells were transfected with a luciferase reporter plasmid driven by a multimerized wild-type (Mef2d wt-Luc) or mutant (Mef2d mut-Luc) MEF2D-binding consensus and luciferase activity was measured. Values are means of three independent experiments. One-way anova, with Tukey’s post-hoc test, ****P *<* 0.0001. **e** Upper panel: analysis of MEF2D phosphorylation by western blot in control (shCTL) and Bδ-KD (shBδ) C2C12 myoblasts at day 3 during differentiation using a phospho-specific antibody against S444 of MEF2D (α-pMEF2D (S444). Total MEF2D (α-MEF2D) was used as loading control. Lower panel: quantification of MEF2D phosphorylation calculated from three experiments as described in **e**, upper panel. Values are mean ± SD. One-sample *t*-test, **P *<* 0.01. ns: not significant. **f** Hierarchical clustering and heatmap of the expression levels (log2 normalized changes) of MEF2-independent (left) and MEF2-dependent (right) genes in control and Bδ-KD C2C12 myoblasts after induction of differentiation for the indicated times

In endothelial cells, a PP2A-Bα holoenzyme dephosphorylates conserved regulatory serines in HDAC7 and promotes its repressive function on transcription^41^. In light of the highly documented roles of HDAC4 and HDAC5, two other members of the class IIa HDACs family in the control of myogenic transcriptional programs^10,42^, we hypothesized that PP2A-Bδ holoenzymes might control HDAC4 and/or HDAC5 repressive function during the fusion process in myoblasts. To test this, we first assess the phosphorylation of class IIa HDACs in myoblasts using an antibody that recognizes a regulatory phospho-serine residue conserved in HDAC4 (Ser246), HDAC5 (Ser259) and HDAC7 (Ser155). We observed a band cross-reacting with the phospho-specific antibody in total myoblast lysates, which we identified as Ser246-phosphorylated HDAC4, based on its apparent molecular weight and its sensitivity to an HDAC4-specific siRNA (Supplementary Fig. 6A, B). In control myoblasts, phosphorylation of HDAC4 Ser246 gradually increased during differentiation (until day 3). At the onset of fusion, HDAC4 phosphorylation decreases to basal levels (Fig. 6a). Phosphorylation of HDAC4 Ser246 was dramatically higher in Bδ-KD cells and did not significantly decrease between days 3 and 6 (Supplementary Fig. 6C), supporting the idea that PP2A-Bδ holoenzymes dephosphorylates HDAC4 primarily during the fusion process. In agreement with this, we also observed that HDAC4, but not HDAC5 specifically interacted with endogenous PP2A-Bδ in C2C12 cells (Fig. 6b).

In muscle cells, HDAC4 primarily represses MEF2-dependent transcription^43^. In agreement with hyperphosphorylation of HDAC4, we observed a reduced interaction between endogenous HDAC4 and MEF2D in Bδ-KD myoblasts at day 3 in DM (Fig. 6c). Concomitantly, MEF2 transcriptional activity was significantly increased as assessed by a MEF2D-reporter assay (Fig. 6d) or hypophosphorylation of its Ser444 (Fig. 6e). To validate this on a genome-wide basis, we performed a series of RNA-seq analyses on control or Bδ-KD myoblasts at days 0, 1, and 3 during differentiation. As expected, the transcriptome of control myoblasts was heavily remodeled during the differentiation process (Supplementary Fig. 6D) with downregulated genes being enriched for biological processes related to cell cycle and cell division (Supplementary Fig. 6E, Clusters #3 and #5) and upregulated genes being enriched for genes involved in skeletal muscle development and contraction (Supplementary Fig. 6E, Clusters #1 and #2). Comparative analysis revealed that, apart from very few exceptions, the expression levels of genes annotated as “skeletal muscle differentiation” (GO:0048741) were not significantly different between control and Bδ-KD myoblasts (Supplementary Fig. 6F), supporting our conclusion that PP2A-Bδ is dispensable for early differentiation of myoblasts. To specifically assess the effects of PP2A-Bδ knockdown on MEF2-regulated genes, we used a previously reported set of 298 MEF2-target genes identified in myoblasts (Supplementary Table 1)^22^. As a control, we selected a set of MEF2-independent genes whose expression was insensitive to MEF2 knockdown (Supplementary Table 1)^22^. Overall, MEF2-independent genes were not affected by PP2A-Bδ knockdown (Fig. 6f). In contrast, a little over 25% of MEF2-dependent genes were significantly increased in Bδ-KD myoblasts (Fig. 6f, clusters #1 and 2). Interestingly, a large proportion of MEF2-dependent genes were not affected (Fig. 6f, clusters #4 and #6) or decreased (Fig. 6f, clusters #3, #5 and #7) following PP2A-Bδ knockdown, suggesting that regulation of HDAC4 by PP2A-Bδ is specific for a subset of MEF2-target genes. This may explain why fusion events but not differentiation events are affected in PP2A-Bδ KD cells.

### PP2A-Bδ controls myoblast fusion through regulation of *ArgBP2* expression

**Fig. 7:**
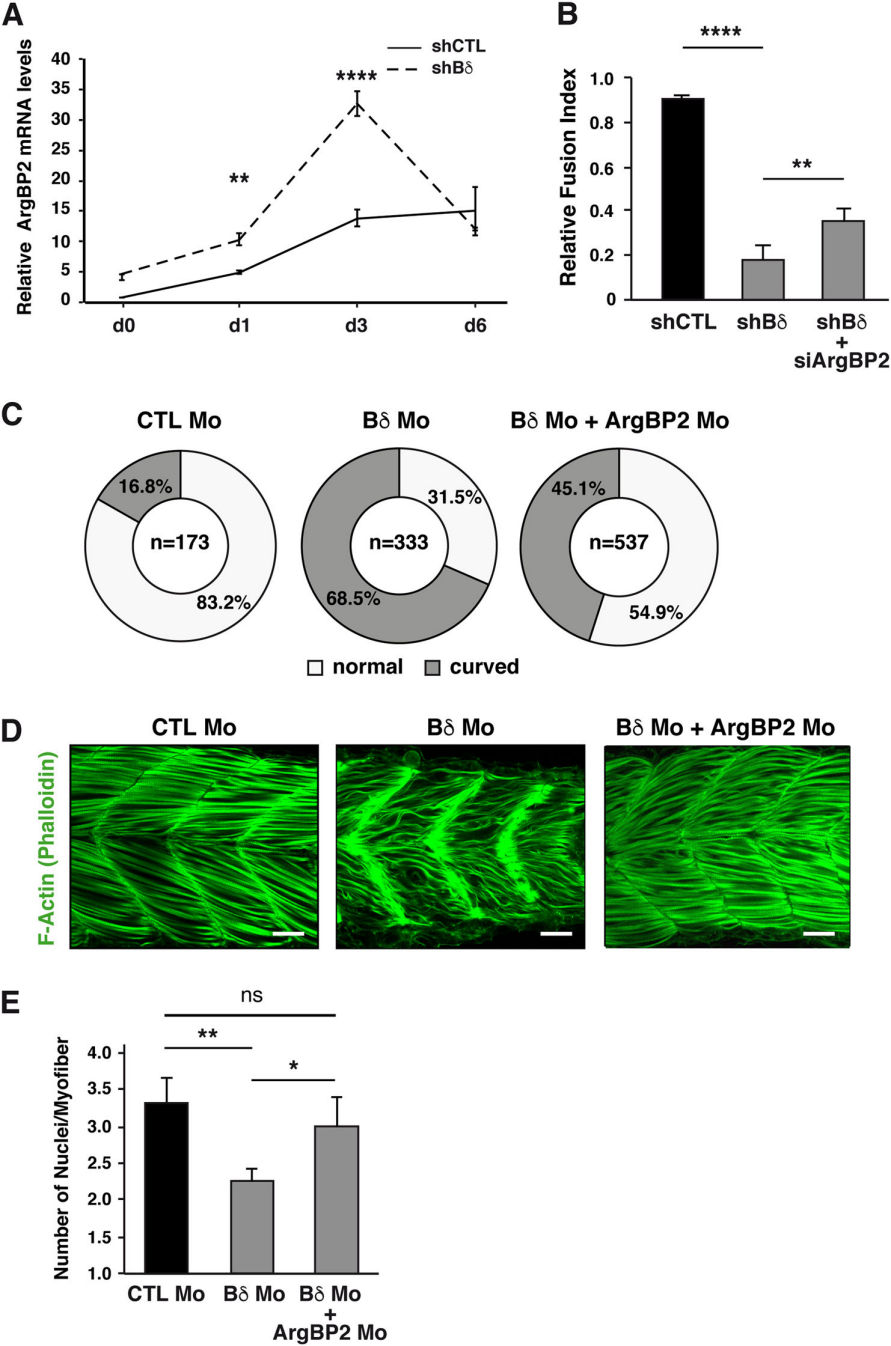
PP2A-Bδ controls myoblast fusion through regulation of *ArgBP2* expression. **a** RT-qPCR analysis of *ArgBP2* mRNA levels in control (shCTL) and Bδ-KD (shBδ) C2C12 myoblasts during differentiation. Values are mean ± SD from three independent experiments. Two-way anova, with Bonferroni correction, ****P *<* 0.0001, **P *<* 0.01. **b** Fusion index of control (shCTL) and Bδ-knocked down (shBδ) C2C12 myoblasts transfected with a control siRNA or an siRNA against *ArgBP2* (siArgBP2) from three independent experiments. One-way anova, with Tukey’s post-hoc test. **P *<* 0.01, ****P *<* 0.001. **c**–**e** Quantification of the curved trunk phenotype (**c**), **d** confocal imaging of F-actin (phalloidin, green, *n* = 10 for CTL Mo and *n* = 15 for Bδ Mo) and **e** quantification of the number of nuclei in fast skeletal myofibers of in 48-hpf zebrafish embryos injected with a control morpholino (CTL Mo, *n* = 5), or with a Bδ morpholino alone (Bδ Mo1, *n* = 3) or together with an ArgBP2 morpholino (Bδ Mo + ArgBP2 Mo, *n* = 10). Scale bars are 25 µm. One-way anova with Tuckey’s post-hoc test, **P *<* 0.01, *P *<* 0.05, ns: not significant

When trying to identify MEF2D-regulated genes that could account for activation of the Abl/CrkII pathway in PP2A-Bδ-depleted cells, we turned our attention to the cytoskeleton adapter protein Arg-binding protein 2 (ArgBP2). Indeed, *ArgBP2*, a well-known inhibitor of Abl and Abl signaling^44^ is a MEF2 target in myoblasts^22^, and was significantly upregulated in Bδ-KD C2C12 (Fig. 7a and Supplementary Fig. 7A). Interestingly, during differentiation of control myoblasts *ArgBP2* mRNA levels follow a kinetics that resembles that of HDAC4 phosphorylation (Figs. 7a and 6a). SiRNA-mediated depletion of HDAC4 but not HDAC5 correlated with increased levels of *ArgBP2* in proliferating myoblasts, indicating that HDAC4 is a bona fide transcriptional repressor of *ArgBP2* expression (Supplementary Fig. 7B). In addition, chromatin immunoprecipitation experiments confirmed that *ArgBP2* is also a MEF2D target in proliferating and differentiating (day 3 in DM) myoblasts (Supplementary Fig. 7C)^22^. These observations indicate that PP2A-Bδ controls expression of *ArgBP2* through the HDAC4/MEF2 axis.

Reducing the expression of ArgBP2 in Bδ-KD cells using an siRNA approach restored (i) normal levels of CrkII Tyr221 phosphorylation, (ii) their ability to elongate (Supplementary Fig. 7D, E) and (iii) their ability to fuse and form myotubes (Fig. 7b). In agreement with these in vitro observations, knocking down *ArgBP2* in Bδ zebrafish morphants significantly reduced the occurrence of the curved phenotype (Fig. 7c), normalized the appearance of actin fibers in fast-twitch muscle (Fig. 7d) and almost completely restored the formation of multinucleated fast myofibers (Fig. 7e, Supplementary Fig. 7F). Altogether, these data establish ArgBP2 as a key effector of PP2A-Bδ-mediated regulation of cytoskeleton rearrangements during myoblast fusion in vitro and in vivo.

## Discussion

Very little information is available about the roles of phosphatases during myoblast differentiation and fusion. Here, we provide the first demonstration that a specific PP2A holoenzyme containing the Bδ regulatory subunit is indispensable for the formation of skeletal muscle myofibers. Through dephosphorylation of HDAC4, PP2A-Bδ controls a set of MEF2-dependent genes required for myocyte fusion. Both in mouse C2C12 myoblasts and in zebrafish, knockdown of PP2A-Bδ specifically interferes with the fusion process without impacting differentiation. This is highly remarkable, considering the wide range of PP2A substrates that have been identified. Our results thus add PP2A-B55δ to the very short list of proteins directly affecting myoblast fusion but not differentiation^45^ and offers unique opportunities to expend our understanding about the signaling pathways behind the fusion process.

To become fusion-competent cells, myoblasts undergo differentiation and characteristic actin-based morphological changes, among which elongation and acquisition of a bipolar shape are the most striking^30^. The absence of PP2A-B55δ in myoblasts prevents the morphogenic events that are necessary for the fusion process^46^. Our study convincingly identified ArgBP2 as a pivotal effector of PP2A-B55δ in the regulation of myoblast morphogenic events prior to fusion. However, it is likely that additional effectors exist among HDAC4-regulated target genes. In addition, we cannot exclude that PP2A-B55δ might also be important for later stages of the fusion process such as cell–cell recognition and adhesion, and pore formation^47^.

**Fig. 8:**
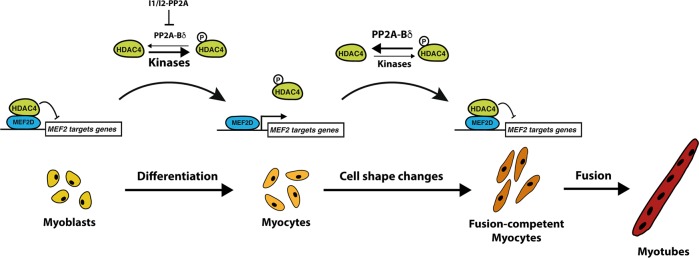
Model of myocyte fusion regulation by PP2A-Bδ. Activation of MEF2-dependent transcription is under the control of reversible phosphorylation of HDAC4 by myogenic kinases and opposing PP2A-Bδ. In proliferating myoblasts, phosphorylation levels of HDAC4 are low because myogenic kinases are inactive. At the onset of differentiation, extracellular myogenic signals activate HDAC4 kinases. Dephosphorylation by PP2A-Bδ is prevented due to high levels of I1/I2PP2A. HDAC4 becomes highly phosphorylated, which promotes its dissociation from MEF2 and activation of MEF2-regulated genes, including effectors of the differentiation programs as well as fusion factors (e.g. ArgBP2). As myocytes complete differentiation, myogenic kinases are inactivated and I1/I2-PP2A levels drop. This allows PP2A-Bδ to efficiently dephosphorylate HDAC4, which reinstates repression over MEF2. Shutdown of MEF2-dependent transcription by PP2A-Bδ terminates the genetic differentiation programs and prevents excessive accumulation of the fusion regulators allowing the morphogenic events required for proper myoblast fusion to occur

Class IIa HDACs, mostly HDAC4 and HDAC5 are considered as master regulators of the genetic programs associated with myoblast differentiation^10^. According to the current model, HDAC4/5-dependent gene repression is achieved through association with MEF2 transcription factors in proliferating myoblasts^4^. For myoblasts to differentiate into myocytes, repression of MEF2-driven gene transcription has to be relieved by signal-dependent phosphorylation of class IIa HDACs, which induces their dissociation from promoter-bound MEF2^10,48,49^. Although our finding that PP2A-B55δ controls HDAC4 repressive activity during myoblast fusion but not differentiation is remarkable, it does not challenge this well-establish paradigm. Instead, we believe that our work brings important missing pieces to the puzzle (Fig. 8). In proliferating myoblasts, class IIa HDACs remain mostly hypophosphorylated, because myogenic kinases are not yet activated. Upon differentiation, class IIa HDAC kinases are activated while PP2A-Bδ remains unable to efficiently dephosphorylate HDAC4 due to high levels of I1/I2PP2A. This tilts the balance towards phosphorylation of HDAC4, thus relieving MEF2D from transcriptional repression and triggering the differentiation gene expression program. At the onset of myocyte fusion, I1/I2PP2A protein levels drop dramatically (Fig. 1d) allowing efficient dephosphorylation of HDAC4 by PP2A-Bδ and subsequent shutdown of specific MEF2-driven genes. This ensures termination of the gene expression programs associated with differentiation, but also precisely regulates expression of factors driving cytoskeletal rearrangements (e.g., ArgBP2), both in terms of timing and level. Therefore, although the HDAC4-MEF2 axis is a key regulator of myoblast differentiation, our findings also suggest that it plays a role in later myogenic events, including fusion. The balance between PP2A-Bδ and HDAC4 myogenic kinases may allow temporal regulation of differentiation and fusion during myogenesis.

During vascular development, we previously reported that B55α-containing holoenzymes specifically act on HDAC7 to control an endothelial gene expression program during vascular lumenogenesis^41^. In HeLa cells, HDAC4 was found in association with PP2A-B56α^50^, while it co-immunoprecipitates with PP2A-B55α in HEK293^51^. Here, we clearly show that PP2A-B55δ binds selectively to HDAC4 in C2C12 myoblasts. Altogether, these observations not only establish class IIa HDACs as main targets for PP2A holoenzymes but they also unravel intricate connections between both protein families. It is very exciting to speculate that specific PP2A holoenzymes might target specific class IIa HDACs in a cellular context-dependent manner. Selective sensitivity of each class IIa HDAC member towards specific PP2A holoenzymes would then be expected to contribute to their remarkable functional specificity during development and cell differentiation^42,52^.

## Disclaimer

The data presented in this manuscript are original and have not been published or submitted elsewhere. All listed authors have approved the manuscript and agreed with the submission.

## Supplementary information

Supplemental Figures and legends

Supplemental Table

Supplementary video 1

Supplementary video 2

Table 1
