# Supplementary material for: Dephosphorylation of HDAC4 by PP2A-Bδ unravels a new role for the HDAC4/MEF2 axis in myoblast fusion
Source: Cell Death Dis. 2019 Jul 4;10(7):512. doi: 10.1038/s41419-019-1743-6 (PMC6609635; doi:10.1038/s41419-019-1743-6)
Supplement: Supplementary file 1 — Supplemental Figures and legends [file 41419_2019_1743_MOESM1_ESM.pdf]

Supplementary Figures and Legends

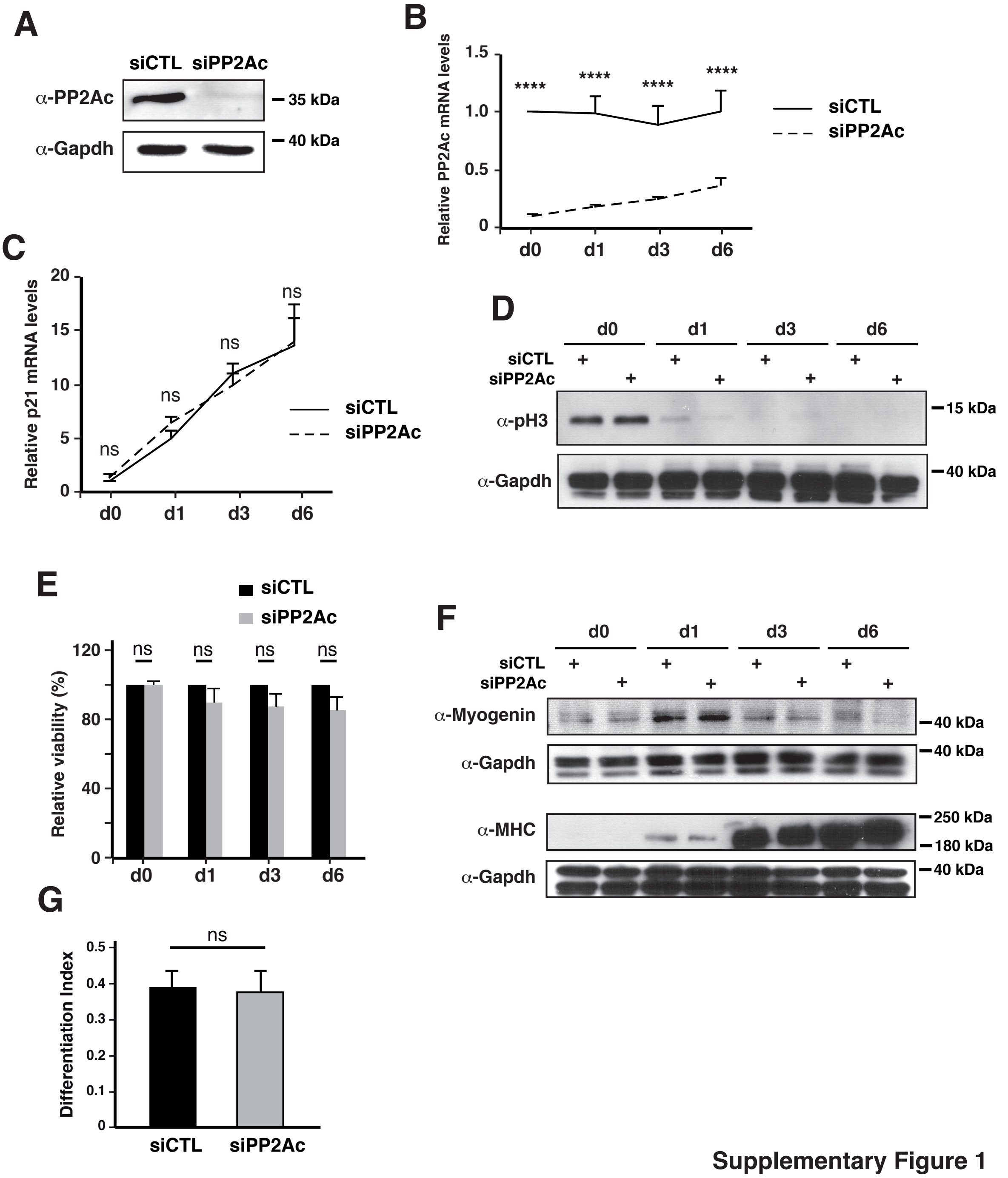

Supplementary Figure 1

Supplementary Figure 1: Knockdown of PP2A catalytic activity does not impact C2C12 proliferation, viability nor differentiation.

(**A**) Western blot analysis (representative of 3 independent experiments) of PP2A catalytic subunit (α-PP2Ac) in C2C12 myoblasts transfected with control siRNA (siCTL) or siRNA against the catalytic subunit of PP2A (siPP2Ac) protein levels at day 1 during differentiation. Levels of GAPDH (α-GAPDH) were used as loading control.

(**B-C**) RT-qPCR analysis of (**B**) PP2A catalytic subunit and (**C**) p21 in differentiating C2C12 myoblasts transfected with a control (siCTL) or a PP2Ac (siPP2Ac) siRNA. Values are mean ± SD from 3 independent experiments. Two-way anova, with Bonferroni correction, **** $P<0.0001$, ns: not significant.

(**D**) Western blot analysis of phospho-Histone H3 (α-pH3) in differentiating C2C12 myoblasts transfected with a control (siCTL) or a PP2Ac (siPP2Ac) siRNA. Levels of GAPDH (α-GAPDH) were used as loading control. Images are representative of 2 independent experiments.

(**E**) Relative cell viability levels assessed by MTS assay in differentiating C2C12 myoblasts transfected with a control (siCTL) or a PP2Ac (siPP2Ac) siRNA. Values are mean ± SD from 4 independent experiments. Two-way anova, with Bonferroni correction, ns: not significant.

(**F**) Western blot analysis of the early differentiation marker myogenin (α-Myogenin) and the late differentiation marker myosin high chain (MHC), in differentiating C2C12 myoblasts transfected with a control (siCTL) or a PP2Ac (siPP2Ac) siRNA. Levels of

GAPDH (α-GAPDH) were used as loading control. Images are representative of 2 independent experiments.

(**G**) Differentiation index at day 6 during differentiation (see methods for details) of C2C12 cells transfected with a control (siCTL) or a PP2Ac (siPP2Ac) siRNA. Values are mean ± SD from 3 independent experiments. Unpaired two-tailed t-test, ns: not significant.

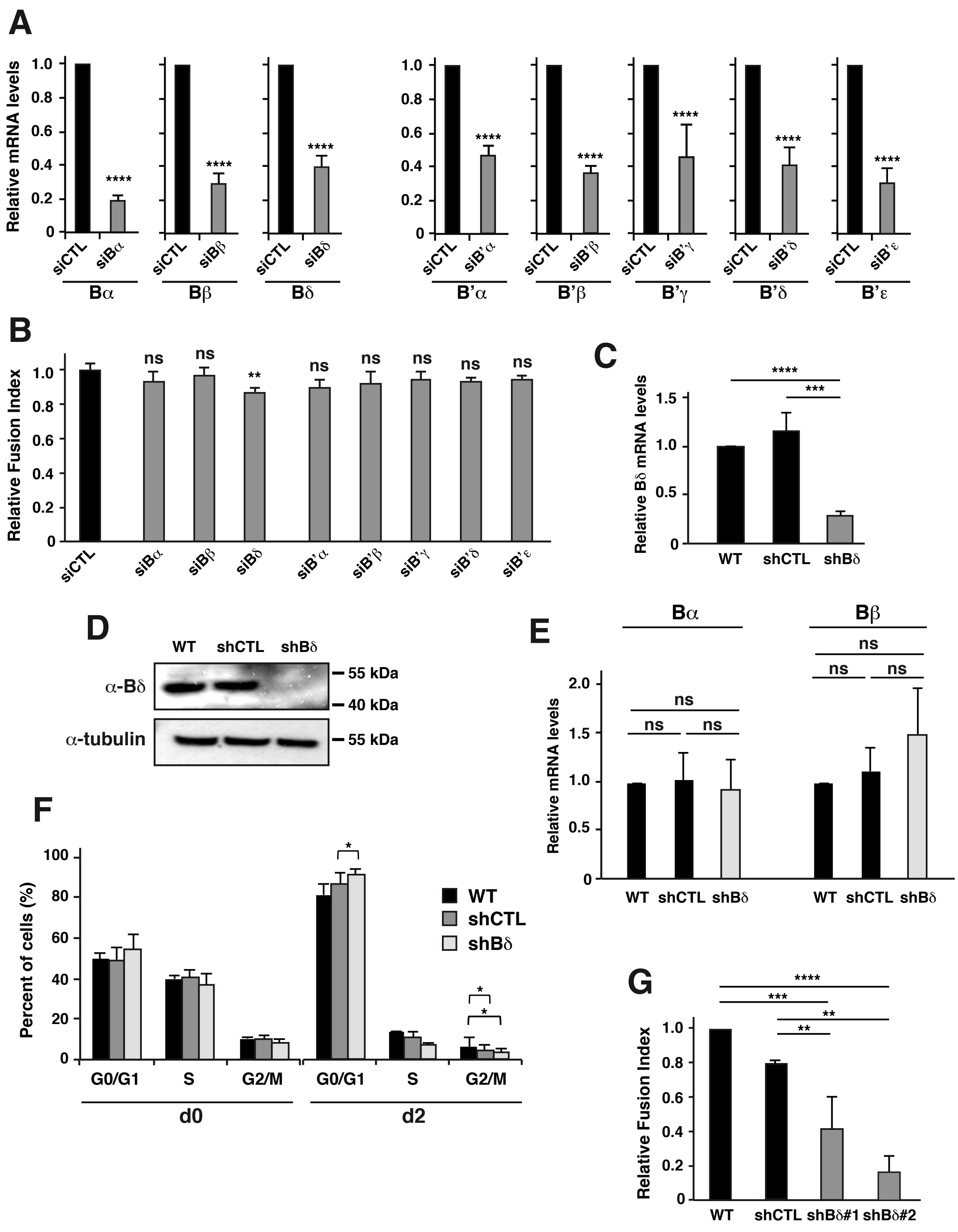

Supplementary Figure 2

Supplementary Figure 2: Knockdown of PP2A-Bδ in C2C12 using siRNA and shRNA approaches.

(**A**) Knockdown efficiencies of siRNA against B- (left panel, siBα, siBβ, siBδ) and B'-(right panel, siB'α, siB'β, siB'δ, siB'γ, siB'ε) subunits in C2C12 cells, as mesured by RT-qPCR and expressed relatively to a control siRNA (siCTL). Values are mean ± SD from 3 independent experiments. One-way anova, with Tukey's post hoc test. **** *P*<0.0001.

(**B**) Fusion indexes of C2C12 cells transfected with the indicated siRNA against B- (left panel, siBα, siBβ, siBδ) and B'-(right panel, siB'α, siB'β, siB'δ, siB'γ, siB'ε) subunits. Results are expressed relatively to the fusion index of C2C12 cells transfected with a control siRNA (siCTL). Values are mean ± SD from at least 3 independent experiments, one-way anova, with Tukey's post hoc test, ** *P*<0.01, ns: not significant.

(**C-D**) (**C**) mRNA and (**D**) protein levels of PP2A-Bδ in wild-type (WT), control (shCTL) and stable Bδ-knocked down (shBδ) C2C12 myoblasts as assessed by qRT-PCR and western blotting, respectively. Tubulin (α-tubulin) was used as loading control for western blot. Values are mean ± SD from at least 3 independent experiments, one-way anova, with Tukey's post hoc test, **** *P*<0.0001, *** *P*<0.001.

(**E**) QRT-PCR analysis of Bα and Bβ levels in wild-type (WT), control (shCTL) and stable Bδ-knocked down (shBδ) C2C12 myoblasts. Values are mean ± SD from at least 4 independent experiments, one-way anova, with Tukey's post hoc test, ns: not significant.

(**F**) Cell cycle analysis of wild-type (WT, black bars), control (shCTL, dark grey bars) and stable Bδ-knocked down (shBδ, light grey bars) C2C12 myoblasts at day 0 and day 2. Percent of cells in G1, S and G2/M are indicated as mean ± SD from 3 independent experiments, one-way anova, with Tukey's post hoc test, * *P*<0.05.

(**G**) Fusion indexes (see methods for details) of wild-type (WT) C2C12 cells or cells transduced by a control (shCTL), or two independent Bδ-specific (shBδ#1 and shBδ#2) shRNA lentiviral vectors. Values are mean ± SD from 3 independent experiments, one-way anova, with Tukey's post hoc test, *** $P<0.001$, ** $P<0.01$.

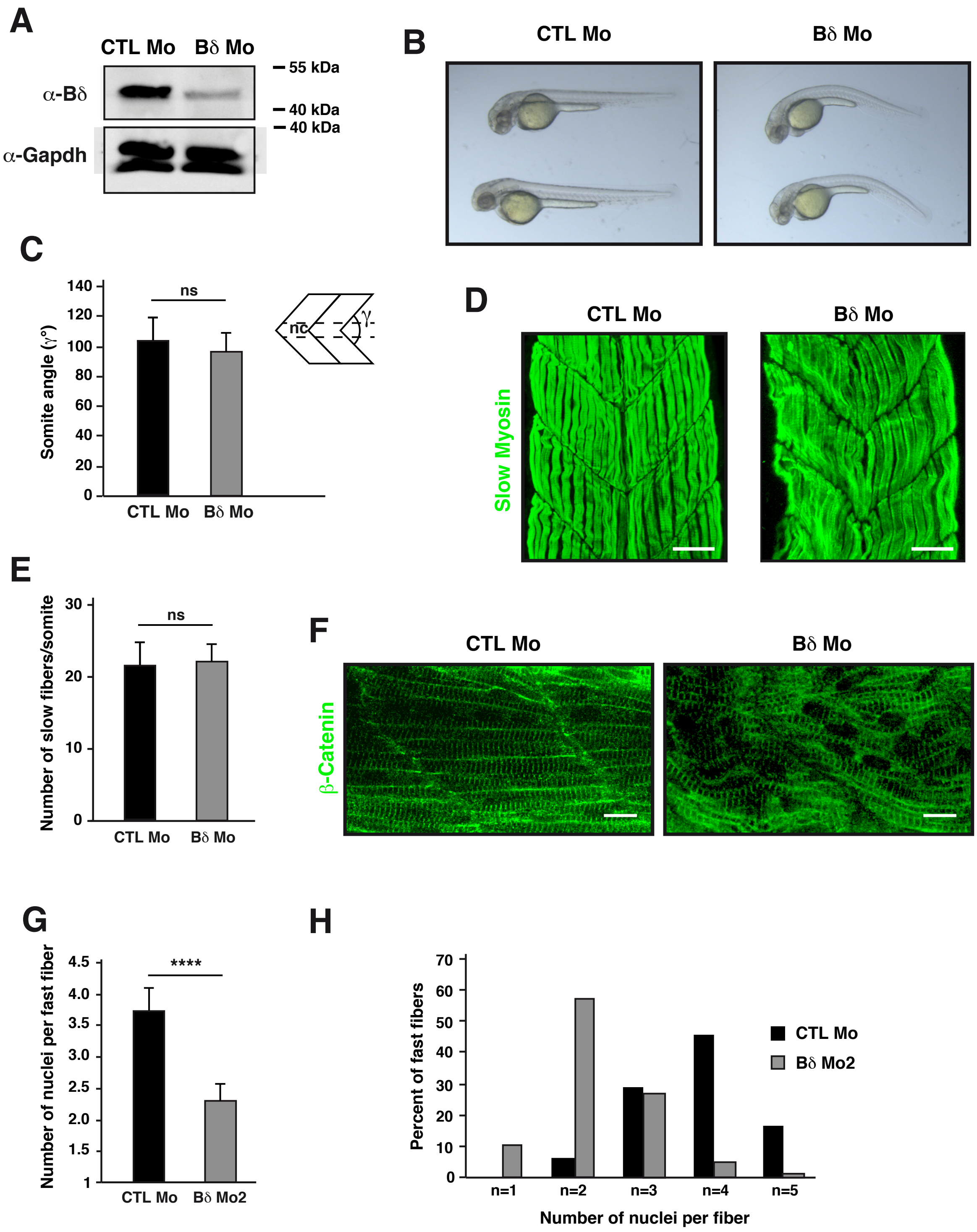

Supplementary Figure 3

Supplementary Figure 3: Knockdown of PP2A-Bδ in zebrafish embryos.

(**A**) Western blot analysis of Bδ (α-Bδ) in 48 hpf zebrafish embryos injected with control morpholino (CTL Mo) and a morpholino targeting zebrafish Bδ orthologue (Bδ Mo). GAPDH (α-GAPDH) was used as loading control. Images are representative experiments from 2 independent experiments.

(**B-E**) (**B**) Bright field pictures representative of at least 10 independent experiments, (**C**) quantification of somite angles (γ; nc: notochord), (**D**) visualization of slow twitch myotome by confocal microscopy and staining for slow skeletal myosin (green) and (**E**) quantification of slow fibers in each somite of 48 hpf zebrafish embryos injected with control morpholino (CTL Mo, n=8 for (C,D) and n=10 for (E)) or a morpholino against Bδ (Bδ Mo, n=17 for (C,D) and n=9 for (E)). Unpaired two-tailed t-test, *** $P<0.001$, ns: not significant.

(**F-H**) (**F**) Confocal pictures of β-catenin (green) highlighting striations, (**G**) quantification of the number of nuclei per fiber and (**H**) proportion of fibers with the indicated number of nuclei in fast myofibers of control (CTL Mo, n=8 for (F) and n=5 for (G,H)) or PP2A-Bδ (Bδ Mo, n=17 for (F) and n=7 for (G,H)) morphant embryos. Unpaired two-tailed t-test, **** $P<0.0001$, ns: not significant.

A

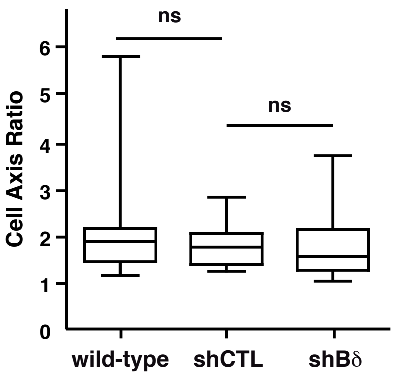

B

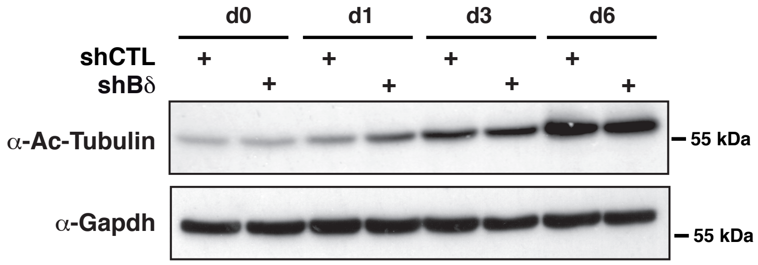

Supplementary Figure 4

Supplementary Figure 4: Knockdown of PP2A-Bδ induces morphogenic defects in C2C12 myoblasts.

(**A**) Major/minor cell axis ratio in wild-type (n=31), control (shCTL, n=16) and Bδ-knocked down (shBδ, n=34) C2C12 myoblasts grown in GM. Values are mean ± SD. Kruskal-Wallis with Dunn's correction. ns: not significant.

(**B**) Western blot analysis of acetylated tubulin (α-Ac-Tubulin) in control (shCTL) and Bδ-knocked down (shBδ) C2C12 myoblasts at the indicated time points during the differentiation process. GAPDH (α-GAPDH) was used as loading control. Images are representative of 2 independent experiments.

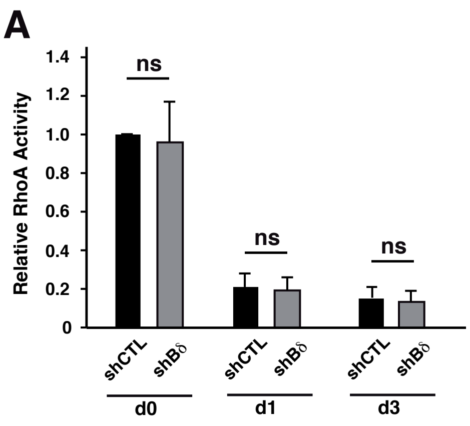

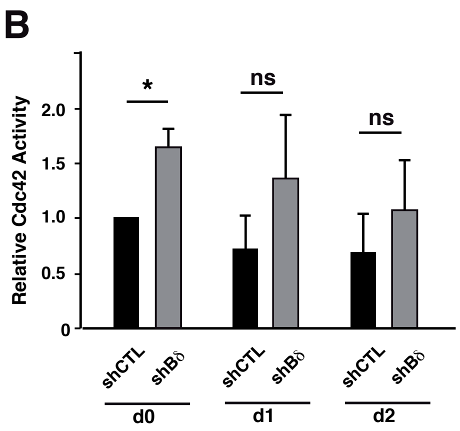

Supplementary Figure 5

Supplementary Figure 5: RhoA and Cdc42 activity in PP2A-Bd KD C2C12.

(**A-B**) (**A**) RhoA and (**B**) Cdc42 activities were measured by GST pull-down assay in control (shCTL) and Bδ-knocked down (shBδ) C2C12 myoblasts at the indicated time points during the differentiation process. Values are mean ± SD from 3 independent experiments, two-way anova, with Bonferroni correction. , ns: not significant

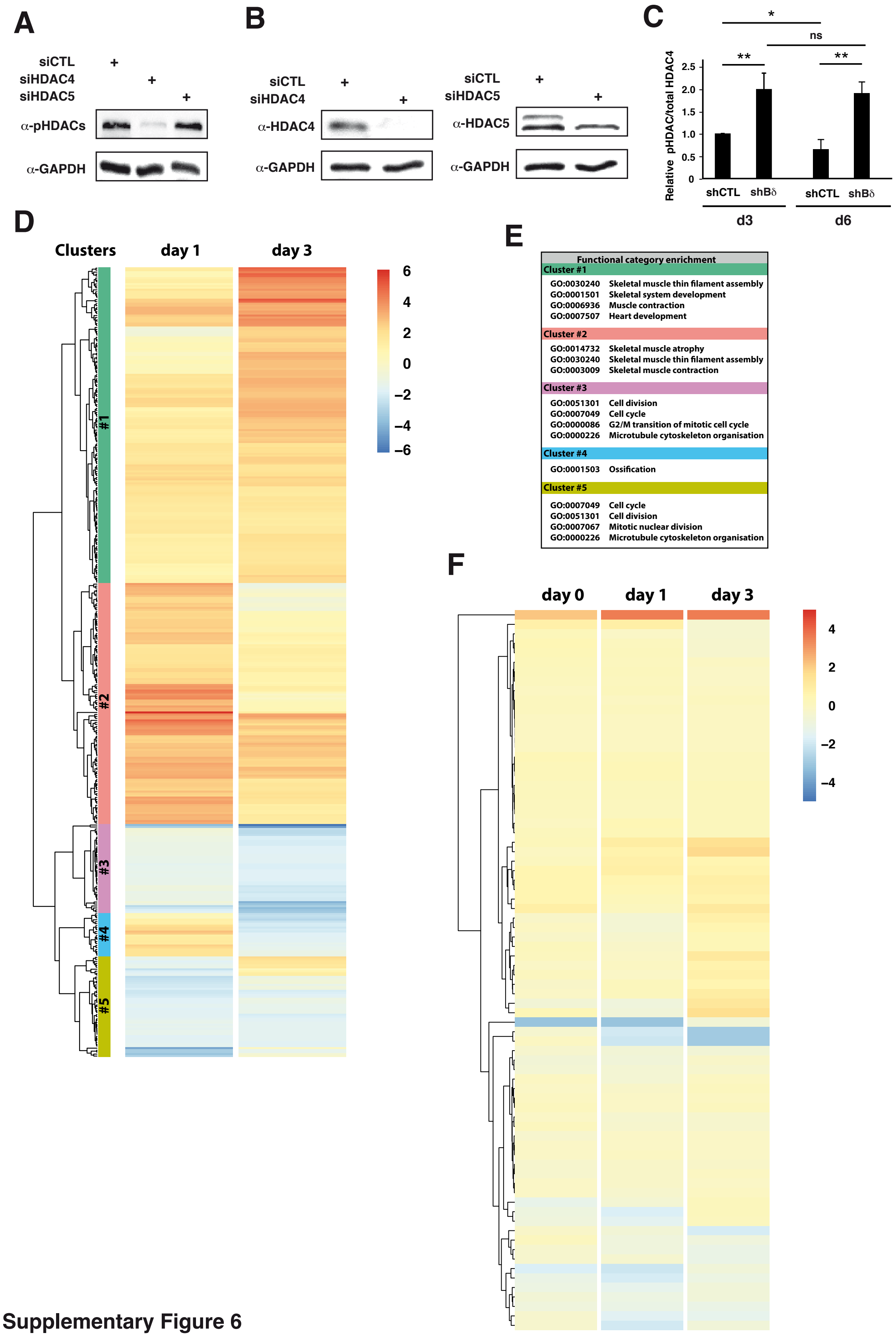

Supplementary Figure 6

Supplementary Figure 6: Knockdown of PP2A-Bδ induces hypophosphorylation of HDAC4 in C2C12 myoblasts.

(**A**) Analysis of class IIa HDAC phophorylation by western blotting using a phospho-specific antibody (α-pHDACs) in C2C12 myoblasts transfected with a control (siCTL), anti-HDAC4 (siHDAC4) or anti-HDAC5 (siHDAC5) siRNA. GAPDH (α-GAPDH) was used as loading control.

(**B**) Western blot analysis of (**left panel**) HDAC4 or (**right panel**) HDAC5 using the corresponding antibody (α-HDAC4 and α-HDAC5, respectively) in C2C12 cells transfected with the indicated siRNA. GAPDH (α-GAPDH) was used as loading control.

(**C**) Quantification of pHDAC4 levels relative to total HDAC4 levels (pHDAC4/HDAC4) in control (shCTL) and Bδ-KD (shBδ) C2C12 myoblasts at day 3 and day 6 during differentiation using a phospho-specific antibody against its S246 (α-pHDAC4 (S246)). Total HDAC4 (α-HDAC4) was used as loading control. Results are means from 3 independent experiments.

(**D**) Hierarchical clustering and heatmap of the expression levels (log2 normalized changes relative to day 0) of genes in control C2C12 myoblasts after induction of differentiation for the indicated times.

(**E**) Top significant GO terms (biological processes) in clusters from (D).

(**E**) Hierarchical clustering and heatmap of the expression levels (log2 normalized changes in Bδ-KD vs control C2C12 myobalsts) of genes annotated as "skeletal muscle differentiation", after induction of differentiation for the indicated times. The list of genes associated with skeletal muscle cell differentiation (GO:0035914), was obtained from Mouse Genome Informatics (http://www.informatics.jax.org/)".

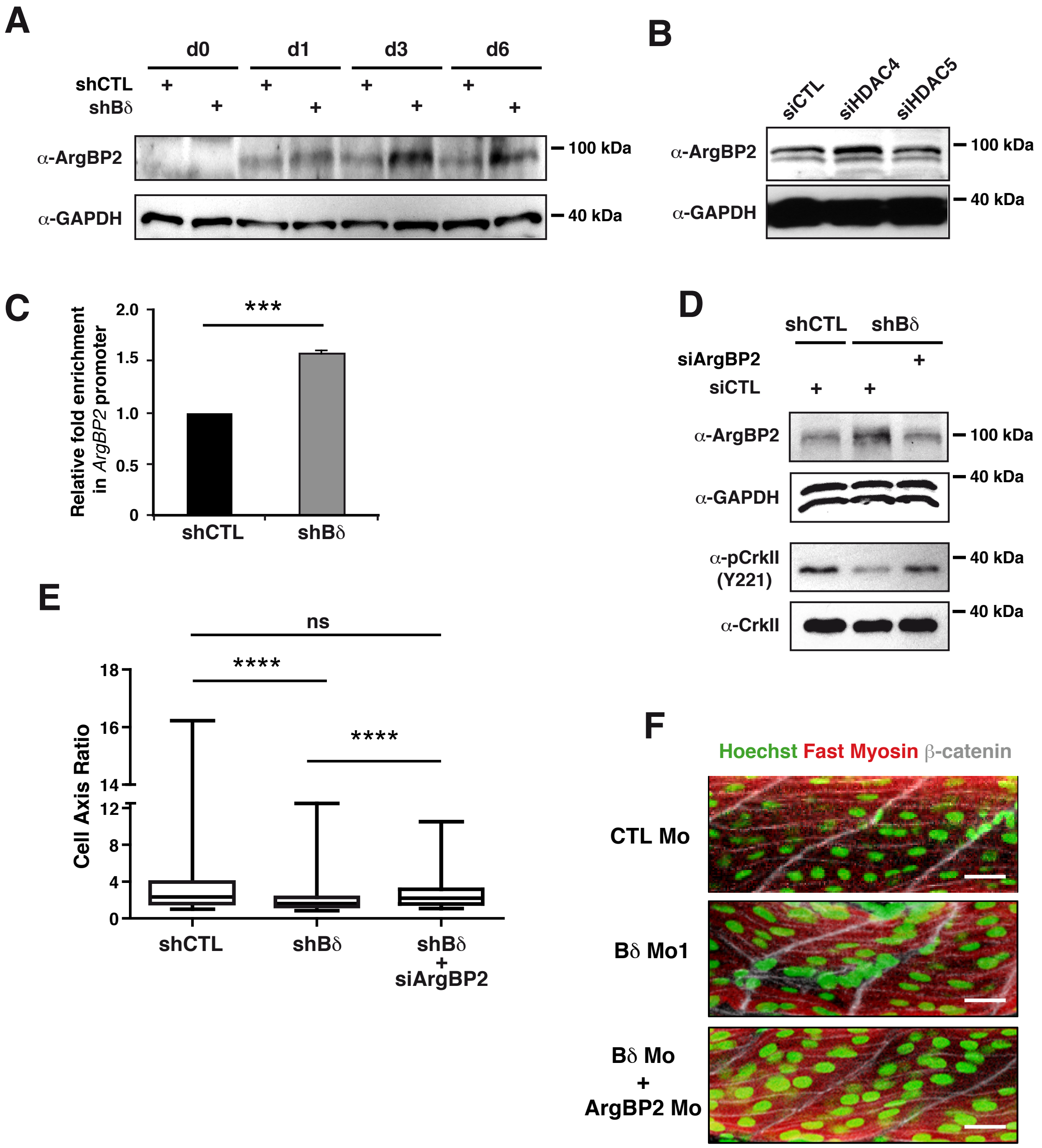

Supplementary Figure 7

Supplementary Figure 7: PP2A-Bδ controls expression of *ArgBP2* via HDAC4.

(**A**) Western blot analysis of *ArgBP2* levels in C2C12 myoblasts transfected with a control (siCTL), anti-HDAC4 (siHDAC4) or anti-HDAC5 (siHDAC5) siRNA. GAPDH (α-GAPDH) was used as loading control.

(**B**) ChIP analysis of MEF2 binding to the *ArgBP2* promoter in control (shCTL) and Bδ-KD (shBδ) C2C12 myoblasts at day 3. Immunoprecipitations were realized with a control IgG or an anti-MEF2 antibody. Results are expressed as mean percent of input ± SD, relative to the control IgG and standardized to shCTL cells, from 3 independent experiments, unpaired two-tailed t-test, *** $P<0.001$.

(**C**) Western blot analysis of ArgBP2 (α-ArgBP2) in control (shCTL) and Bδ-knocked down (shBδ) C2C12 myoblasts at the indicated time points during the differentiation process. GAPDH (α-GAPDH) was used as loading control. Images are representative of 2 independent experiments.

(**D**) Analysis of ArgBP2 (α-ArgBP2) and activity of CrkII as assessed by phosphorylation of its inhibitory Y221 (α-pCrkII (Y221)) in control (shCTL) and Bδ-knocked down (shBδ) C2C12 myoblasts transfected with a control siRNA or a siRNA against *ArgBP2* (siArgBP2). GAPDH (α-GAPDH) and total CrkII (α-CrkII) were used as loading control.

(**E**) Major/minor cell axis ratio in control (shCTL) and Bδ-knocked down (shBδ) C2C12 myoblasts grown in GM and transfected with a control siRNA or an siRNA against *ArgBP2* (siArgBP2). Results were calculated on >200 cells/experiment from 2 independent experiments, unpaired two-tailed t-test, **** $P<0.0001$.

(**F**) Representative confocal pictures of fast skeletal myosin (red) and β-catenin (white) in control (CTL Mo, n=6 independent experiments) or PP2A-Bδ (Bδ Mo, n=6

independent experiments) morphant embryos, injected with morpholino against ArgBP2 (ArgBP2 Mo, n=10 independent experiments). Nuclei of fast skeletal fibers were stained with Hoechst (green). Scale bars are 100µm.
